# Little Cigars are More Toxic than Cigarettes and Uniquely Change the Airway Gene and Protein Expression

**DOI:** 10.1038/srep46239

**Published:** 2017-04-27

**Authors:** Arunava Ghosh, Sabri H. Abdelwahab, Steven L. Reeber, Boris Reidel, Abigail J. Marklew, Andrew J. Garrison, Shernita Lee, Hong Dang, Amy H. Herring, Gary L. Glish, Mehmet Kesimer, Robert Tarran

**Affiliations:** 1 Marsico Lung Institute, The University of North Carolina at Chapel Hill, NC, 27599, USA; 2 Department of Pathology and Laboratory Medicine, The University of North Carolina at Chapel Hill, NC, 27599, USA; 3 Department of Chemistry, The University of North Carolina at Chapel Hill, NC, 27599, USA; 4 Department of Biostatistics, The University of North Carolina at Chapel Hill, NC, 27599, USA; 5 Department of Cell Biology & Physiology, The University of North Carolina at Chapel Hill, NC, 27599, USA

## Abstract

Little cigars (LCs) are regulated differently than cigarettes, allowing them to be potentially targeted at youth/young adults. We exposed human bronchial epithelial cultures (HBECs) to air or whole tobacco smoke from cigarettes vs. LCs. Chronic smoke exposure increased the number of dead cells, lactate dehydrogenase release, and interleukin-8 (IL-8) secretion and decreased apical cilia, cystic fibrosis transmembrane conductance regulator (CFTR) protein levels, and transepithelial resistance. These adverse effects were significantly greater in LC-exposed HBECs than cigarette exposed cultures. LC-exposure also elicited unique gene expression changes and altered the proteomic profiles of airway apical secretions compared to cigarette-exposed HBECs. Gas chromatography-mass spectrometry (GC-MS) analysis indicated that LCs produced more chemicals than cigarettes, suggesting that the increased chemical load of LCs may be the cause of the greater toxicity. This is the first study of the biological effects of LCs on pulmonary epithelia and our observations strongly suggest that LCs pose a more severe danger to human health than cigarettes.

Tobacco smoke is inhaled by 1.3 billion people worldwide and remains a major risk factor for several diseases including many types of cancer, cardiovascular disease and chronic obstructive pulmonary disease (COPD)^1^. In the US alone, 42 million people smoke tobacco and this has accounted for ~20 million premature deaths since the publication of the first Surgeon General’s report in 1964^1^. COPD is currently the third leading cause of mortality worldwide^2^ and is characterized by “persistent airflow limitation associated with a heightened chronic inflammatory response in the pulmonary tissue^3^. The two major components of COPD are chronic bronchitis and emphysema, with the former being “clinically and epidemiologically” more relevant^4^. The airway surface liquid (ASL) is the first point of contact of inhaled tobacco smoke with the lung. A well-hydrated ASL is required to maintain mucus clearance, a key component of the lung’s innate defense system. The ASL also contains over 1000 proteins including mucins, anti-microbial peptides/proteins, and proteases that are required for innate defense and the appropriate regulation of inflammation^5^.

The occurrence of COPD is directly related to tobacco smoking^3^. Mucus dehydration, mucus hypersecretion, and chronic inflammation are hallmarks of the chronic bronchitis component of COPD and they all contribute to the progressive/irreversible airflow limitation, increased chances of infection, and lung destruction seen with this disease^6^. The airway hydration status plays a critical role in maintaining the sterility of lung by mucociliary clearance and prevention of infection^7^. The CFTR is a cAMP-regulated anion channel whose function is absolutely required for ASL hydration and to maintain mucociliary clearance and the sterility of lungs, as indicated by the genetic disease cystic fibrosis^8^. In cystic fibrosis, a lack of functional CFTR leads to severe mucus stasis/dehydration, inflammation and infection. It has been demonstrated that exposure to cigarette smoke causes CFTR inhibition in humans and ASL dehydration *in vitro* that is caused by a reduction in the CFTR protein^9^,^10^,^11^. Smoke from other tobacco products is predicted to exert similar effects on CFTR, but this hypothesis has not yet been tested.

Cigarettes are defined as tobacco wrapped in paper and LCs are tobacco wrapped in tobacco leaf and weighing <3 lbs per 1000. Until recently, LCs have historically evaded the FDA’s bans on advertising and flavorings, making them more appealing to youth and young adults^12^. Despite little being known about the health effects of LCs, these products are perceived as less harmful than cigarettes by young adults^13^. Since the airways are directly exposed to inhaled tobacco smoke, they bear the brunt of the immediate effect of inhalation^14^. In part, the detrimental effects of tobacco exposure are counteracted by the lung’s innate defense system that works to limit the damages caused by smoke by providing anti-oxidants and by removing inhaled toxicants^15^. While cigarette smoke exposure has been extensively studied, other tobacco products such as LCs have not been studied and may pose hitherto unrecognized threats to pulmonary health. Studies of assessment of harm are therefore important in order to guide the FDA and other regulatory bodies to draft legislation and to inform the public. In this study, we exposed primary, well-differentiated HBECs to air and tobacco smoke from Kentucky research cigarettes, and LCs to evaluate their relative effects on airway epithelial viability/function.

## Results

### LCs cause significantly greater adverse effects on airway epithelial function than cigarettes

**Figure 1:**
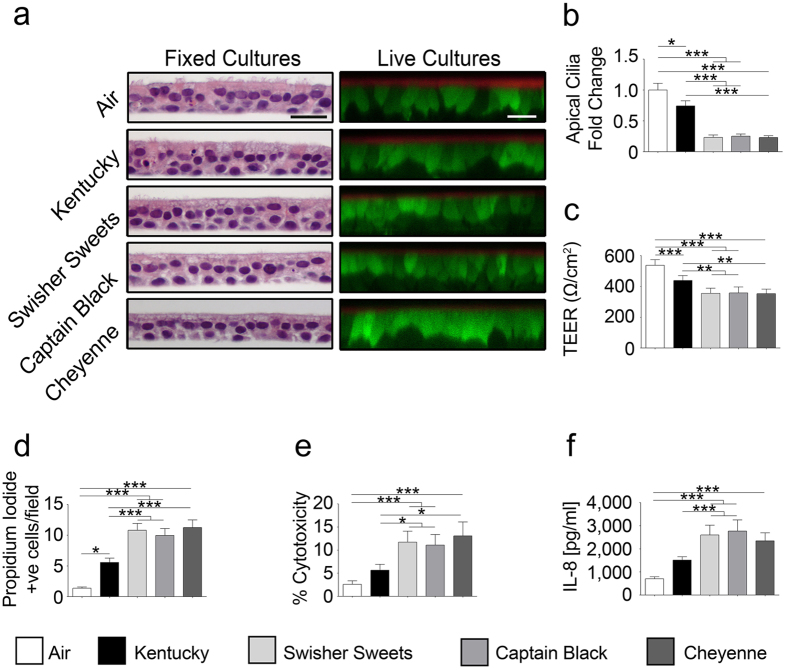
Chronic tobacco smoke causes enhanced cytotoxicity and inflammation. (**a**) Left, light micrographs showing H&E stained human airway cultures and right, XZ confocal micrographs showing calcein-AM (green) and rhodamine dextran (red) stained live cells and airway surface liquid respectively. Bar graphs show mean changes as noted for (**b**) apical cilia abundance (n = 10), (**c**) transepithelial electrical resistance (n = 14) and (**d**) propidium iodide (PI) positive cells (n = 23), (**e**) percent cytotoxicity measured by percentage of LDH release (n = 20) and (**f**) IL-8 release (n = 20) into basolateral media following chronic smoke exposure (*p < 0.05, **p < 0.001, ***p < 0.0001).

To assess the impact of LCs on human airway epithelia, we chronically exposed HBECs and (i) fixed cells to perform gross analysis by light microscopy and (ii) imaged live cells by XZ confocal microscopy. Chronic tobacco smoke exposure from either cigarettes or LCs did not cause gross cellular abnormalities and the cultures remained viable throughout the exposure period, as indicated by calcein-AM uptake and the persistence of a thin film of ASL (Fig. 1a). However, apical ciliary abundance was markedly decreased in smoked cultures (Fig. 1a,b and Supplementary Fig. 1a). Surprisingly LC exposure resulted in a significantly greater decline in cilia abundance than Kentucky or air exposure. Similarly, LC-exposed HBECs exhibited a significantly greater decrease in transepithelial electrical resistance than Kentucky or air exposed HBECs (Fig. 1c).

### Chronic LC exposure causes cytotoxic and pro-inflammatory effects

**Figure 2:**
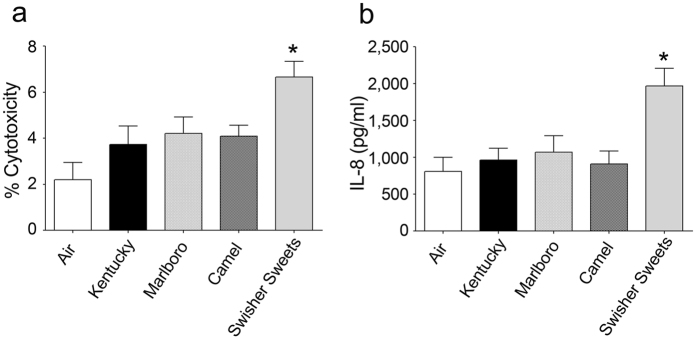
Little cigar smoke exposure causes more cytotoxicity and inflammation than commercial cigarettes. HBECs were chronically exposed to 10 × 35 ml puffs of smoke per day for five days from Kentucky, Marlboro, Camel cigarettes and Swisher Sweets little cigar. (**a**) LDH release as a marker of cytotoxicity and (**b**) and IL-8 release into the basal media. ^*^Denotes p < 0.05 different to air. All data points represent n = 6.

We next stained cells with propidium iodide to determine the numbers of dead cells per epithelia. We observed a significant increase (p < 0.05) in propidium iodide positive cells in chronic Kentucky cigarette-exposed HBECs (Fig. 1d and Supplementary Fig. 1b). However, LC exposure caused a significantly greater (p < 0.001) increase in the numbers of propidium iodide positive cells per culture (Fig. 1d and Supplementary Fig. 1b). The total number of dead cells was less than 5% per field, which is likely why the epithelia were able to maintain a thin film of ASL and a significant transepithelial resistance. Lactate dehydrogenase (LDH) is released from cells when their plasma membranes are damaged and provides a sensitive way to evaluate cytotoxicity^16^. Chronic tobacco smoke exposure revealed a significant increase in LDH release compared to air-exposed cells for all groups (Fig. 1e). Again however, LDH release was significantly greater (p < 0.05) following LC exposure than for Kentucky exposure (Fig. 1e). Cigarette smoke induces IL-8 secretion from pulmonary epithelia, which is notable because IL-8 is a chemoattractant that is responsible for neutrophil infiltration^17^. Chronic LC exposure led to significantly increased IL-8 secretion in media that was significantly greater than air or Kentucky exposures (Fig. 1f). During the smoking regimen used in Fig. 1, we smoked the whole LC or the whole cigarette, which yielded different numbers of puffs (18 vs 14 respectively). Therefore, we further evaluated the effects of 10 equal puffs- from Kentucky cigarettes, Swisher Sweets and two commercial cigarette brands (Marlboro and Camel; Fig. 2). Despite generating equal numbers of puffs, the chosen biomarkers of exposure, i.e. percent cytotoxicity (LDH release) and IL-8 secretion were still significantly greater after Swisher Sweets exposure than for any tested cigarette brand (Fig. 2).

### Chronic LC exposure decreases CFTR protein levels and decreases ASL height to a greater extent than cigarettes

**Figure 3:**
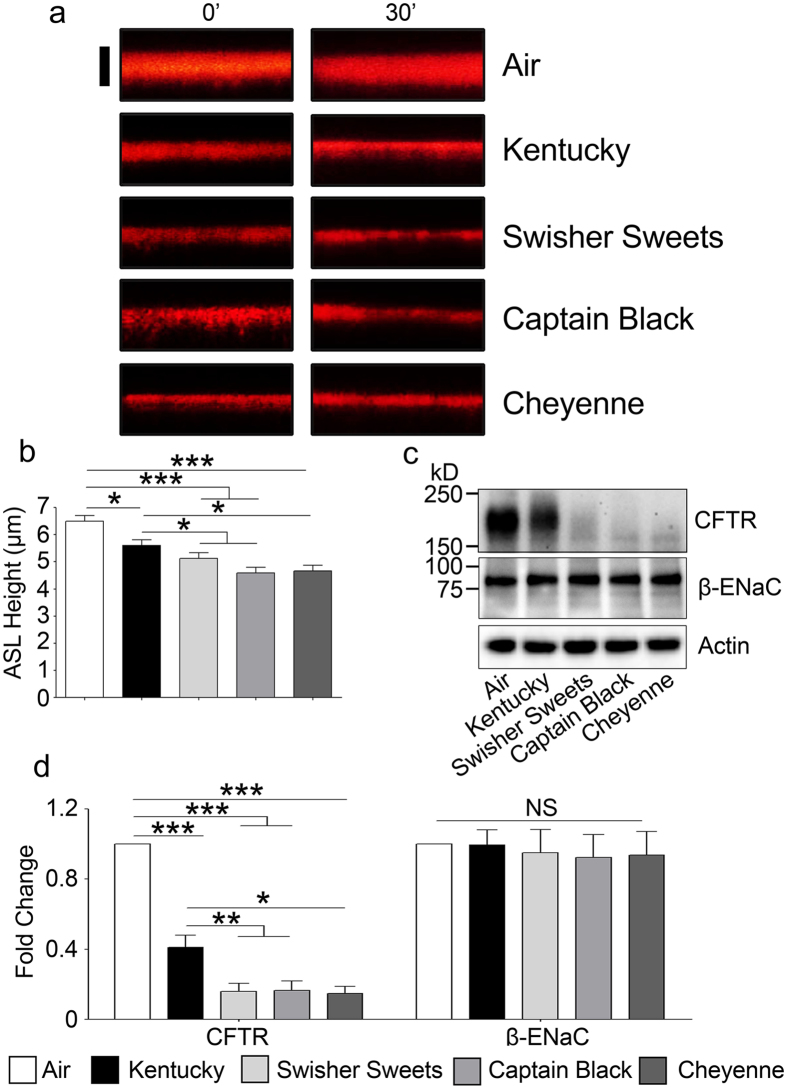
Chronic LC exposure induces significantly greater airway surface liquid (ASL) dehydration than cigarette exposure. (**a**) Representative XZ confocal micrographs of ASL (red) labeled with rhodamine-dextran. Scale bar is 10 μm. 0′ denotes ASL before fifth day exposure and 30′ indicates ASL after 30 minutes of fifth day exposure. (**b**) Mean ASL height measured by XZ confocal microscopy after chronic (5 day) exposure to air, Kentucky cigarettes or LCs (n = 10). (**c**) HBECs were exposed to smoke or air for 5 days, lysed and Western blots were then run and probed for CFTR, β-ENaC and actin as indicated. Bands corresponding to the relevant protein sizes (kD) are shown in the figure. (**d**) Mean integrated densitometry for CFTR and β-ENaC respectively after normalization to the actin loading control (n = 10 blots). (*p < 0.05, **p < 0.001, ***p < 0.0001).

We and others have previously shown that acute and chronic cigarette smoke exposures decrease CFTR protein levels *in vivo* and *in vitro*, leading to a reduction in ASL volume in HBECs^9^,^11^. Such a decrease in ASL hydration is predicted to contribute to the mucus dehydration/plugging seen in COPD patients. Thus, we assayed the effects of chronic LC smoke exposure on CFTR and ASL height using the five day smoking protocol described above. Five days of chronic Kentucky cigarette smoke exposure caused a significant decrease in ASL height (Fig. 3a,b). However, the decrease in ASL height was significantly greater for all 3 LC (Fig. 3a,b). Chronic tobacco exposure also decreased CFTR protein levels and consistent with the greater decrease in ASL height seen with LC exposure, CFTR levels were also more greatly decreased after chronic LC smoke exposures than after air or Kentucky cigarette smoke exposure (Fig. 3c,d). In contrast, neither actin, which we used as a loading control, nor the epithelial sodium channel beta subunit (β-ENaC), were decreased after exposure to any tobacco product (Fig. 3c,d), suggesting that this effect was specific for CFTR.

### Chronic LC smoke exposure induces expression of unique and more genes than Kentucky cigarette smoke exposure

**Figure 4:**
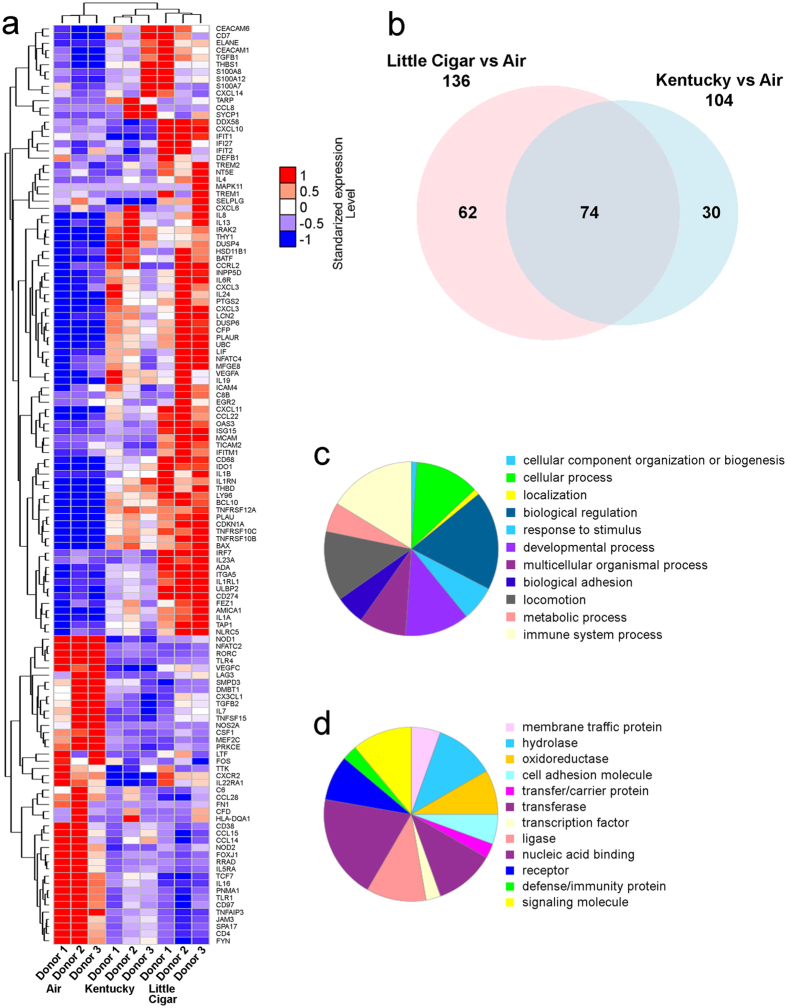
Chronic LC exposure results in unique changes in gene expression. HBECs derived from 3 individual donors were exposed to chronic smoke from Kentucky cigarettes vs. Swisher Sweets LCs for 5 days vs. air controls and probed with the Nanostring pan-cancer-immune gene panel. (**a**) Heat map showing genes altered across the groups (q-value <0.01, fold change >2). Color codes represent normalized expression levels of the genes after standardization across samples with mean = 0 and standard deviation = 1. (**b**) Venn diagram comparing the number of altered genes in the tobacco-exposed groups relative to air exposure (q-value <0.1, fold change > 2). (**c**) Pie chart representing altered genes representing biological processes following LC smoke compared to Kentucky cigarette exposure. (**d**) Pie chart representing protein classes corresponding to altered genes following LC smoke compared to Kentucky cigarette exposure.

To determine whether the increased changes wrought by LCs extended beyond the functional measures described in Figs 1 and 2, we chronically exposed HBECs to Swisher Sweets LCs, Kentucky cigarettes or air (control) for 5 days, lysed cells to obtain RNA and then probed the samples with the NanoString PanCancer Immune panel of 770 genes. A comparative analysis of the gene expression patterns indicated that exposure to Swisher Sweets LCs altered significantly more genes than Kentucky cigarettes (Fig. 4a,b). A total of 136 genes were differentially expressed in the LC-exposed group compared to air. However, 74 genes were altered in both smoke exposure groups and LCs altered 62 unique genes (Fig. 4b). In contrast, Kentucky cigarette exposure altered 104 genes, of which 30 were unique (Fig. 4b). The full list of significantly altered genes is shown in Supplementary Tables 1–3. A network analysis of altered genes indicated more complex interactions in HBECs exposed to LCs than HBECs exposed to cigarettes (Supplementary Figs 2 and 3). Furthermore, while the clustering coefficient remained same for both exposure groups (0.659 for air vs Kentucky and 0.644 for air vs LC), both the number of edges and the average node degree were increased following LC smoke exposure (318 and 6.06 respectively) compared to Kentucky smoke exposure (177 and 4.6 respectively; Supplementary Figs 2 and 3). Pathway analysis further revealed that LC exposure changed pathways involved in cellular functions, biological regulation, responses to stimuli, localization and immune responses (Fig. 4c). LC exposure also caused significant alterations in protein groups involved in membrane trafficking, immunity, signalling molecules, cell adhesion molecules, nucleic acid binding, hydrolase and transferase activity (Fig. 4d), again suggesting that the resultant phenotype following chronic LC exposure is different to that seen with cigarette exposure.

### Chronic LC smoke exposure significantly alters the ASL proteome

**Figure 5:**
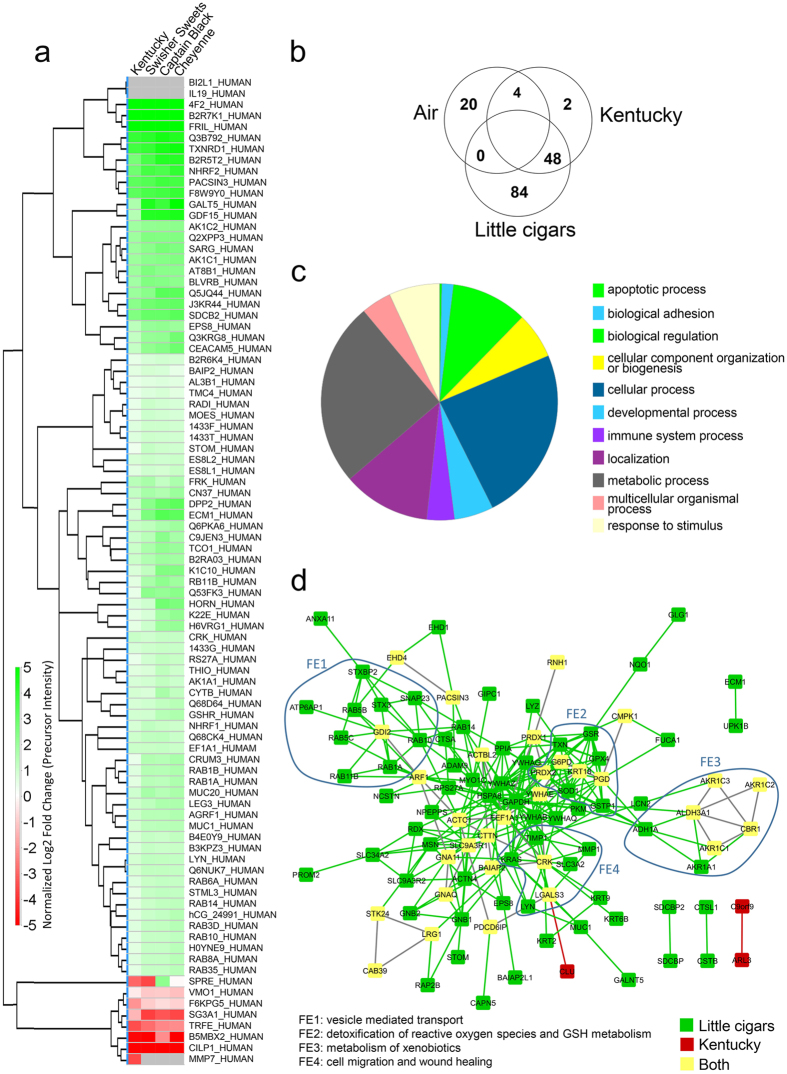
LC smoke exposure causes greater changes to the ASL proteome than cigarette smoke exposure. (**a**) Heat map of significantly changed proteins relative to air controls. Significance was set at p ≤ 0.001. (**b**) Venn diagram showing proteins that are upregulated in each group. (**c**) Pie chart representing the biological process classification for significantly changing proteins of all exposure groups. (**d**) Reactome map showing the functional enrichment (FE) of proteins with significant increases after Kentucky cigarette and LC exposure.

Since ASL is important for innate defense of the lung^6^, we next used a proteomics approach to comprehensively measure biomarkers of LC exposure. In the proteomic analysis of secretions collected from chronically tobacco smoke exposed HBECs, ~200,000 spectra were acquired, leading to the identification of ~4000 peptides which could be assigned to 1300 proteins. ~930 of these proteins were assigned at least 2 peptide identifications and subsequently included in the label-free proteomic quantitation using total intensities as the ssums of individual precursor peak areas. A complete list of all proteins used in the quantification can be found in Supplementary Table 4. Changes in expression of proteins displaying an ANOVA p-value below 0.001 are shown in a heat map subsequent to chronic tobacco exposure and using these criteria, 134 proteins were altered after chronic tobacco exposure (Fig. 5a). Consistent with the increased biological effects seen with LC exposure (Fig. 1), 84 proteins were significantly and uniquely upregulated after LC exposure while only 2 proteins were uniquely altered after cigarette exposure (Fig. 5b). As shown in Fig. 4c, the vast majority of proteins were involved in cellular or metabolic process. However, proteins involved in the immune response and apoptosis were also altered. We next performed pathway analysis on these proteins. This analysis revealed that vesicle-mediated transport, detoxification of reactive oxygen species, metabolism of xenobiotics and cell migration/wound healing pathways were all altered by LC exposure (Fig. 5d), suggesting that HBECs upregulate proteins to compensate for the increased toxic burden of LC exposure.

### Mass spectrometric analysis of tar particle extracts reveal greater numbers of chemicals in tobacco smoke than Kentucky cigarettes

**Figure 6:**
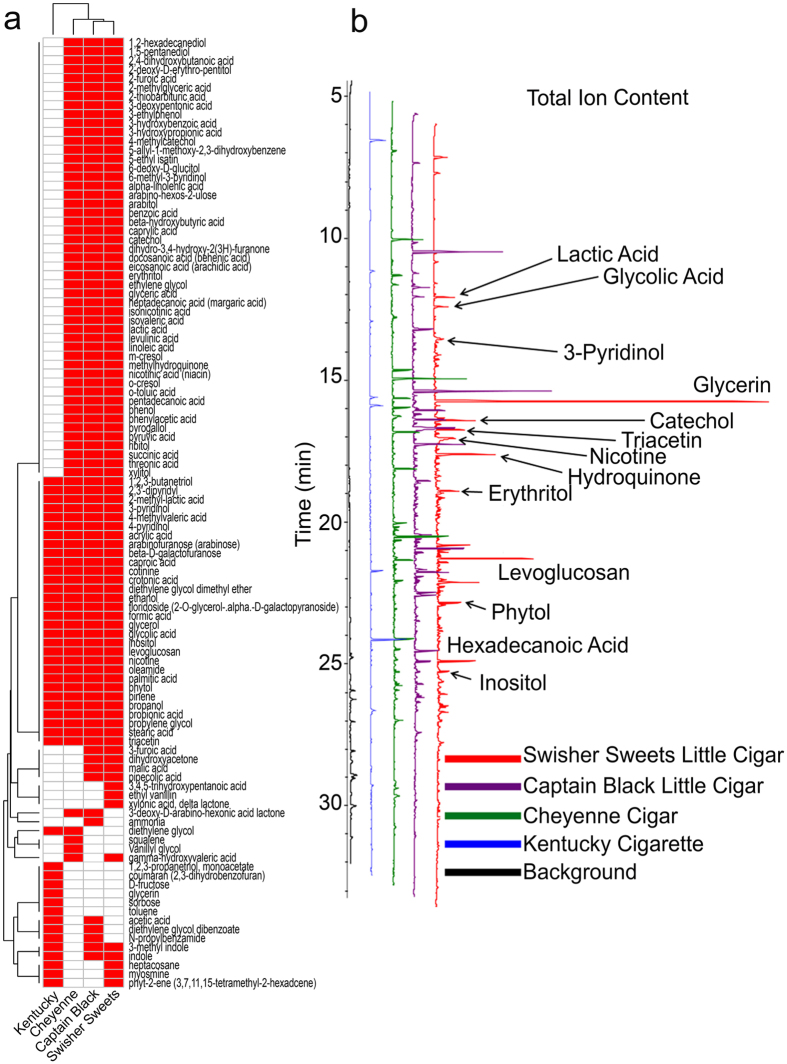
LC combustion yields different chemicals than cigarette combustion. (**a**) Heat map showing presence (red) or absence (white) of chemical compounds from mass spectrometry of tar particles in different tobacco products. (**b**) Gas Chromatogram showing peaks representing different compounds in the collected tar phase from different tobacco products.

**Figure 7:**
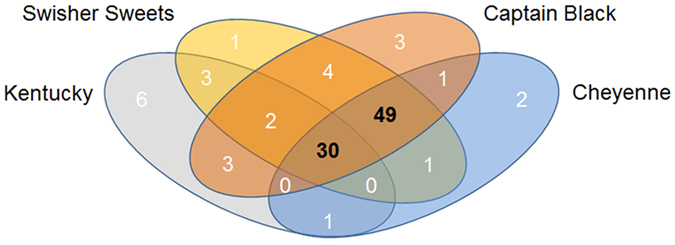
Little cigars have more chemical constituents in their tar phase than Kentucky research cigarettes. Whole tobacco smoke tar particles were analyzed by GC-MS and 30 chemicals were found to be common to all the tobacco products. 49 unique chemical entities were identified in tar particles from all 3 little cigars. In contrast, 6 unique chemicals were identified in Kentucky cigarettes.

Tobacco smoke is a complex and dynamic mixture of thousands of compounds including several carcinogens and oxidants^18^. To see if the increased toxicity of LCs correlated with an increased chemical output, we evaluated the tar particle phase of LCs and Kentucky cigarettes. LC deposited significantly more tar particles per puff on Cambridge filter pads than Kentucky cigarette (Supplementary Fig. 4). We then collected the tar particles from whole tobacco smoke and solubilized them in methanol to comprehensively evaluate all detectable chemical entities as a measure of relative chemical deposition using GC-MS. This analysis revealed that extracts from LC tar particles displayed a markedly different chemical profile to Kentucky cigarettes. Gas chromatography mass spectra indicated that compounds present in all tobacco products were found in higher quantities in LCs relative to Kentucky cigarettes (Fig. 6a,b). Additionally, unique 49 compounds were identified in LCs that were absent from Kentucky cigarettes (Fig. 7 and Supplementary Table 5).

## Discussion

Cigarettes are known to be highly toxic and can exert multiple effects on the pulmonary system, including, but not limited to, increases in apoptosis, inflammation, protease activation, DNA breaks as well as deranging the mucus clearance system^6^. However, whilst the biological effects of cigarettes have been well studied, next to nothing is known about the effects of LC exposure to the lung. Thus, whilst secondhand smoke from LCs impairs arterial flow-mediated dilation in rats^19^, the potential for LCs to induce harm in humans remains undetermined. Using surface airway epithelia obtained directly from human lungs, we found that LCs exerted greater cytotoxicity and pro-inflammatory cytokine secretion and wrought greater changes at both the gene and protein levels. Thus, based on our data, we propose that cigarette exposure cannot be used as a proxy for LC exposure and that LCs need to be evaluated independently.

Our primary HBECs can be cultured for ~2 months *ex vivo* and have similar levels of ion transport and ciliation as airway epithelia *in vivo*^20^. Furthermore, HBECs have previously been shown to be predictive of outcomes in both cystic fibrosis patients and smokers^21^, suggesting that their use is valid. For example, ion transport rapidly declines after 10 puffs of tobacco smoke *in vitro* and *in vivo*^9^ and hypertonic saline elicits a more durable effect on mucus clearance rates in cystic fibrosis patients than normal subjects that is mirrored in HBECs^21^. Our exposure dose was set so as not to be 100% toxic and to not kill the cells regardless of tobacco type. Indeed, epithelia remained intact and with a thin film of ASL, suggesting that they were still viable and capable of some ion/fluid transport, even after chronic tobacco smoke exposure (Fig. 1a). Airway epithelia expand ~35% of their energy to move ions, so bioelectric properties can be used as a biomarker of cell viability^22^. Consistent with these observations, the transepithelial electrical resistance declined by ~20%, but remained significantly high to facilitate vectorial ion/water transport. Schamberger *et al*. have previously demonstrated that cigarette smoke exposure decreases zonula occludens (ZO)-1 and ZO-2 proteins, leading to the disruption of epithelial tight junction integrity, which may be the cause of our observed decrease in transepithelial electrical resistance^23^. Of note, Knowles *et al*. reported that the basal potential difference was decreased by ~20% in smokers, which using Ohm’s law, they attributed to a ~20% decrease in the transepithelial electrical resistance^24^. Thus, our 5 day exposure induces similar biologic changes in ion transport as seen *in vivo* for smokers. Similar with our previous studies^9^, we observed a significant decrease in ASL height after the 5 day exposure (Fig. 3a,b). Based on our Western blot data, these decreases in ASL height were likely due to reductions in CFTR levels in the face of continued ENaC activity (Fig. 3c,d). As with the transepithelial electrical resistance data (Fig. 1), we observed a significantly greater decrease in CFTR protein levels and ASL height for the LCs than for Kentucky cigarettes (Fig. 3c,d). Given that a reduction in mature CFTR protein levels has extensively been linked to airway disease^6^ that LCs cause a greater decrease in CFTR levels should be taken very seriously and indeed suggests that chronic LC use may have a greater propensity for causing lung pathology.

We also observed a significant decrease in cilia abundance (Fig. 1b), a phenomenon that has previously been detected in smokers^25^. Like the reduction in ASL height, decreased cilia function is indicative of impaired mucus clearance and impending lung disease, and may be a precursor to airways remodeling^6^. The degree of deciliation was significantly greater after LC exposure than for cigarette smoke exposure, likely indicating a greater toxic burden induced by LCs. We also measured additional biomarkers of exposure/toxicity following the chronic 5 day smoke protocol. For example, propidium iodide binds to DNA, and can only get access to cells when they are damaged, and LDH is only released from damaged cells. For both measures of cytotoxicity, the effects were significantly greater for LCs (Fig. 1d,e). Chronic neutrophilia is a hallmark of chronic bronchitis, and the subsequent release of neutrophil elastase contributes to lung damage^26^. IL-8 is a cytokine that is involved in neutrophil recruitment/migration into the lungs and interleukin secretion was significantly greater after LC than Kentucky cigarette exposure, suggesting that in addition to being more toxic, that LCs may also induce more inflammation and place LC smokers at greater risk from chronic neutrophilia^17^,^27^.

Gene analysis using the NanoString PanCancer Immune panel revealed that LCs altered a significantly larger number of genes than cigarettes (Fig. 4a–d and Supplementary Tables 1 and 2). Tobacco smoke exposure has previously been shown to alter several genes in both HBECs and *in vivo* in health smokers^28^. Here, we found that 74 genes were commonly altered after both LC or cigarette smoke exposure (Fig. 4a,b). However, an additional 62 unique genes were altered by chronic LC exposure (Fig. 4b and Supplementary Table 3). Of these genes, it is interesting that FOXJ1, which stimulates ciliogenesis^29^ was significantly downregulated after LC exposure, which is consistent with the deciliation seen in Fig. 1. Similarly, consistent with the increase in cytokine secretion observed in Fig. 1, many cytokines and cytokine receptors were altered following LC exposure (Supplementary Table 1) and in some cases, uniquely altered following LC exposure (Supplementary Table 3). Of note, kinases such as JAK3 and MAPK11, which facilitate the development of inflammation^30^ were uniquely upregulated after LC exposure. Taken together, these unique gene changes are strongly suggestive of an increased inflammatory burden after LC exposure that may be associated with greater airways remodeling.

The ASL is in direct contact with tobacco smoke and represents an easily accessible pool of proteins that are absolutely required for lung health and can be used as biomarkers of exposure/toxicity. Overall, we reliably identified ~900 proteins in the ASL (Fig. 5). Consistent with the effects seen on cellular toxicity, ASL proteome changes were significantly greater with LCs than Kentucky cigarettes (Fig. 5). It is interesting that many of the altered proteins were involved in key pathways that would be used to ameliorate the increased toxic burden, including proteins involved in the detoxification of xenobiotics, e.g. aldehyde dehydrogenase (ALDH3A1)^31^ or carbonyl reductase (CBR1)^32^ and proteins like peroxiredoxin (PRDX2) and glutathione reductase (GSR)^33^ that are required to metabolize reactive oxygen species. Reactive oxygen species are thought to contribute to the toxicity of tobacco exposure^34^ and upregulation of these proteins is likely a contributory response to this process. Given that the ASL is involved in innate defense and that we observed dysregulation of innate defense proteins, our data suggest that pulmonary defenses may be altered by tobacco exposure. Indeed, tobacco smokers are thought to be immunocompromised and less able to deal with both viral and bacterial infections and our data suggest that LC users may be more compromised than cigarette users^35^. However, further epidemiological data will be needed to confirm or refute these observations. It is important to note that the ASL proteome *in vitro* is derived from a relatively pure population of bronchial epithelial cells whilst *in vivo*, alveolar epithelia, macrophages, and glandular secretions all contribute to the ASL proteome. Thus, an advantage of our preparation is that we can definitively say that LC exposure significantly altered the airway epithelial proteome. However, the effect of LCs on other pulmonary cell types will need to be determined and likely, greater differences will be observed in samples derived directly from patients. Importantly, our results suggest that LCs should not be considered a “safer alternative” to cigarettes.

Previous reports indicated that LC smoke contains greater amounts of toxic chemicals compared to lung compared to cigarette smoke^36^. However, these studies were limited to the elemental composition. Whilst LCs generate more tar per unit than Kentucky, equal puffs of LCs still resulted in significantly increased toxicity (Fig. 2). Furthermore, using a highly sensitive GC-MS technique, we identified a large number of previously undescribed compounds that were present in tar extract from LCs but not Kentucky cigarettes (Figs 6 and 7). 48 unique chemical entities were identified in LC smoke tar particles and while the effects of these many of these compounds on the lung are not fully understood some have been studied. For example, 4-methylcatechol induces carcinogenecity in rats^37^ and has been linked to cytotoxicity^38^. Furthermore, benzoic acid derivatives induce differentiation of cancer cell lines^39^ and catechol estrogens have been shown to act as initiators of cancer^40^. Thus, we speculate that the increased tar produced (Supplementary Fig. 4) along with the greater chemical diversity generated by LCs (Figs 6 and 7) may cause the increase in toxicity (Figs 1, 2 and 3) and drive the changes in the genome/ASL proteome (Figs 4 and 5).

LC sales have been increasing, especially amongst youth and young adults, and they are seen as a cheap alternative to cigarettes since they are taxed at a lower rate^41^. Because of the perception of reduced risk from LCs use and the availability of different flavors, LCs have thus gained popularity amongst younger smokers^42^. Flavors are thought to help with the initiation of smoking by masking the harsh taste of tobacco, thus facilitating addiction^43^. Although the use of these products amongst younger smokers has been well documented^44^, these products have historically not been regulated as strictly as cigarettes, in part due to a lack of information regarding their effects on health. Thus, our data may (i) provide further data to help regulate these products and (ii) raise awareness that LCs are not safer than cigarettes and in fact, may be more toxic. Collectively our data indicate that LCs exert significantly more toxic effects than regular cigarettes and elicit a greater biological response from the epithelia as they adapt to the noxious environment caused by chronic tobacco exposure. The distinctly different chemical makeup of LCs and their more severe effects suggest that strict regulation on sale, use and advertisement of these products for the sake of general public health safety effected.

## Methods

### Human bronchial epithelial cell isolation and culture

Human bronchial epithelia were isolated from main stem/lobar bronchi of human excess donor and excised recipient normal lung by enzymatic digestion following protocol #03–1396 approved by the University of North Carolina Biomedical Institutional Review Board^45^. All methods were performed following relevant guidelines and regulations. Informed consent was obtained from all donors or authorized representatives of the donors. Cultures were used after 3–6 weeks of seeding.

### Chronic whole tobacco smoke exposure

Unless otherwise noted, tobacco smoke was generated according to ISO standards using a Borgwaldt LC1 smoke engine and applied to cells that were located in a specially designed smoke chamber^9^. For all studies, we used 1 × 35 ml puff per 30 second using a butt length of 36 mm. Tobacco smoke was generated using identical parameters by either an LM1 or LX1 smoke engine (Borgwaldt). We tested 3 commercial tobacco products: The brand names were Swisher Sweets, weight 1.34 ± 0.01 gm, length 9.9 cm per unit; Captain Black, weight 1.13 ± 0.01 g, length 9.8 cm per unit and Cheyenne cigars (Full Flavor), weight 1.38 ± 0.02 g, length 9.8 cm per unit. For comparison, we generated smoke from Kentucky Research Cigarettes (code 3R4F, Class A cigarettes, weight 1.04 ± 0.01 g, 8.4 cm per unit). For air exposure, cells were exposed to 18 air puffs that was equivalent to the mean number of puffs obtained from LCs. HBECs were placed in sterile Ringer’s solution (in mM) 120 NaCl, 5.2 KCl, 1.2 MgCl_2_, 1.2 CaCl_2_.2H_2_O, 12 NaHCO_3_, 24 HEPES, 10 glucose, pH 7.4 during the exposure. The cultures were subsequently placed in culture media and returned to the 5% CO_2_ at 37 ˚C incubator. Cells were exposed to smoke from 1 cigarette or LC every day vs. air control for 5 days and washed with PBS 1 h after exposure, with daily changes of serosal media, keeping the cultures sterile for the entire 5 day exposure.

### Apical cilia detection

Following chronic tobacco exposure, HBECs were washed with PBS and fixed with 4% paraformaldehyde for 15 min on ice. Cultures were washed with PBS to remove paraformaldehyde and blocked with 5% FBS for 1 h. HBECs were then incubated overnight with a primary antibody raised in rabbit against amino acids 427–441 amino acid residues of human α-tubulin (Rockland antibodies and assays, 600-401-880) to stain surface apical cilia and followed by staining with goat anti rabbit Dylight 649 secondary antibody (Vector laboratories, DI-1649) and DAPI. Mean fluorescence was recorded on a multi-plate reader (Infinite Pro, Tecan) and representative images were captured on a Leica SP8 confocal microscope with a 40X dry objective.

### Histology

HBECs were washed both mucosally and serosally with PBS 1 h after the 5th day of exposure and fixed in 10% neutral buffered formalin bilaterally. HBECs were embedded in paraffin and standard H&E staining was performed. Images were captured on a Nikon Microphot-SA microscope with a 50X oil objective using Nikon DXm1200 digital camera.

### LDH assay

LDH release was measured following manufacturer’s instructions. 24 h after the last exposure HBEC mucosal surfaces were lavaged mucosally with 200 μl PBS. HBECs were then incubated at 37 °C for 30 min, the lavage was removed, centrifuged at 4,000 x G for 5 min at 4 °C and the supernatant collected. Serosal media was collected and centrifuged in the similar way. Then, HBECs were lysed with 100 μl of lysis buffer (50 mM Tris, 150 mM NaCl, 1% NP-40, pH 7.4) with protease inhibitors (cOmplete, EDTA-free; Roche Applied Science) on ice for 15 min. The lysate was centrifuged at 10,000 × G for 10 minutes at 4 °C and the supernatant was assessed for total LDH activity using a Cytotoxicity Detection Kit (Roche) following manufacturer’s instructions. Percent cytotoxicity was calculated as previously described^46^.

### IL-8 ELISA

Serosal media were obtained 24 h after the last chronic air or tobacco smoke exposure and centrifuged at 4,000 × G for 5 min at 4 °C and supernatant stored immediately at −80 °C for subsequent analysis. The IL-8 concentration in the serosal media was measured by DuoSet^®^ Human CXCL8/IL-8 Kit (R&D Systems) following the manufacturer’s instructions.

### Transepithelial electrical resistance measurements

Transepithelial electrical resistance was measured using an EVOM machine (World Precision Instruments) using an STX2 electrode as per manufacturer’s instructions as described^47^.

### Live dead cell staining

After 1 h of tobacco exposure, cells were washed with PBS and stained with 3 μM Calcein AM (Life Technologies) in PBS mucosally for 30 min at 37 °C. Cells were then washed with PBS again and incubated with 150 μM Propidium Iodide (Sigma) for 15 min at 37 °C to stain the dead cells. Cells were washed with PBS again and 10 random images per culture were captured using a Leica SP5 confocal microscope with a 63X glycerol immersion. On average, each field had approx. 220–230 cells.

### XZ-confocal microscopy of airway surface liquid height

To measure the height of the ASL, PBS (20 μl) containing 2 mg/ml rhodamine-dextran (10 kDa; ThermoFisher) was added to cultures 1 h before exposure. Excess fluid was removed from the cultures after incubation for 15 minutes at 5% CO_2_/37 °C. 10 predetermined points were XZ scanned using an automated stage on a confocal microscope (Leica SP8; glycerol 63 x immersion lens) as described^48^. Cultures were returned to the incubator between time points. For all studies, perfluorocarbon (PFC) (FC-77; 3 M) was added mucosally during imaging to prevent evaporation of the ASL.

### Western blotting

After 1 h of WTS exposure, HBECs were washed with ice cold PBS and lysed with buffer containing 25 mM Tris, 150 mM NaCl, 1% NP-40, 1% Sodium deoxycholate pH 7.4, protease inhibitors (cOmplete, EDTA-free; Roche Applied Science) on ice for 15 min with occasional shaking. The lysate was centrifuged at 5,000 x G for 10 minutes at 4 °C and the supernatant was used for western blotting. After using the BCA assay to determine protein concentration, 50 μg of protein was heated at 37 °C for 30 min and resolved using 4–15% Bio-Rad Mini-Protean TGX gels, transferred to PVDF membrane. Membranes were blocked with 5% NFDM in Tris Buffered Saline with 0.2% Tween-20 (TBST) for 2 h at room temperature followed by primary antibody incubation overnight at 4 °C in 5% NFDM in TBST (1:5000 596 CFTR antibody that recognizes CFTR residues 1204–1211; Cystic Fibrosis Foundation Therapeutics Inc.). Membranes were probed with HRP conjugated secondary antibodies (Jackson ImmunoResearch Laboratories Inc.) and developed using BioRad Clarity western ECL substrate using ChemiDoc^TM^ MP Imaging system (Bio-Rad). Blots were stripped and probed for α-actin (anti α-actin antibody, clone EP184E, Millipore) (1:2000).

### Gene expression and pathway analysis for chronic smoke exposure

To analyse changes in gene expression, passage 1 HBECs derived from 3 different donors were plated on 12 mm transwell-clear inserts and cultured for 1 month under air-liquid interface conditions (i.e. until fully differentiated). Cultures were then exposed to 14 × 35 ml puffs from 1 cigarette or cigar per day for 5 days, with puffs of air serving as the control. During the exposure, basolateral perfusion was maintained with culture media (DMEM high glucose:LHC basal media, 1:1 ratio) at 37 °C and 5% CO_2_. 1 h after smoke exposure on the 5^th^ day, cultures were exposed to RLT plus buffer (Qiagen) and frozen at −80 °C. RNA was isolated using RNeasy kit (Qiagen) following the manufacturer’s protocol. Samples were probed with the PanCancer Immune panel (NanoString) which is a 770-plex gene expression panel and analysed with the nCounter analysis system (NanoString) to evaluate gene expression. Differential expression analysis from nCounter counts were performed using the Bioconductor R package, NanoStringDiff, with donor code and exposure as independent variables^49^. Differentially-expressed genes were determined based on FDR adjusted q-values ≤0.1 and ≥2 fold change for both Kentucky exposure and LC exposure compared to air exposure for the Venn diagram and Supplementary Tables 1–3. For the heat map (Fig. 3a), we only showed genes with ≥2 fold change and q-values ≤0.01 due to space constraints. The gene networks were generated by STRING v10.0 using estimated mean values from the differential expression analysis output of NanoString nCounter (NanoString)^50^. Biological processes and protein classes altered in the different groups were analysed using the Panther classification system^51^.

### Proteomic analysis of apical secretions of chronically tobacco exposed airway cultures

Apical secretions were collected and processed to identify and quantitate the proteins using mass spectrometry based proteomic analysis^52^. The raw data was processed and searched against the Uniprot protein database (Homo sapiens) using the Proteome Discoverer 1.4 (Thermo Scientific) software. Parameters used in the Sequest search engine: 10 ppm mass accuracy for parent ions and 0.02 Da accuracy for fragment ions, with 2 missed cleavages allowed. Carbamidomethyl modification for cysteines was set to “fixed” and methionine oxidation to “variable”. Scaffold 4.4.8 (Proteome Software Inc.) was used to validate MS/MS based peptide and protein identifications. Peptide identifications were accepted if they could be established at greater than 95.0% probability by the Scaffold Local FDR algorithm. Protein identifications were accepted if they could be established at greater than 95.0% probability and contained at least 2 identified peptides. Protein probabilities were assigned by the Protein Prophet algorithm^53^. Proteins that contained similar peptides and could not be differentiated based on MS/MS analysis alone were grouped to satisfy the principles of parsimony. Proteins were annotated with GO terms from gene_association.goa_human^54^. Protein quantification and generation of the heatmap was performed using perSPECtives 2.0.6 (Proteome Software Inc.) by summarizing the intensities of identified precursor ions for each protein as protein intensities. The protein intensities were normalized to total intensity of all identified proteins in each sample. Statistical significance between the air and smoke exposure groups were determined by ANOVA. For quantification, the average protein intensities for 6 cell culture replicates were compared to determine protein fold changes. The Reactome analysis of proteins was performed using the STRING algorithm^50^. Overlay analysis of the two resulting reactome networks was prepared in the software Cytoscape V3.4.0 using the DyNet application^55^.

### Gas chromatography-mass spectrometry (GC-MS)

The samples were eluted from Cambridge filters in 5 mL of methanol. The filters were placed in glass vials, methanol was added, and they were vortexed for 1 minute, followed by filtration through a 0.22 μm filter. The methanol was evaporated under a stream of nitrogen, and 300 μL 99:1 BSTFA:TMCS and 200 uL pyridine was added. They were loosely capped and placed in a 40 °C water bath for 2 hours, then stored at −20 °C until analysis. Analysis was performed on a HP 5890 gas chromatograph with mass spectrometry detection. 1 μL injections were made onto a DB-5 capillary column. The temperature was ramped from 60 °C to 300 °C at 10 degrees per minute. Helium was used as carrier gas. Compounds eluting from the column were ionized by electron ionization and analyzed using a standard HP MSD mass spectrometer. Data analysis was performed with the AMDIS software tool, developed by NIST and available for free at http://chemdata.nist.gov/mass-spc/amdis/downloads/ using the NIST 2008 EI MS database.

### Statistical analysis

For analysis of the effects of chronic smoke exposure, data for Swisher Sweets and Captain Black were pooled and treated as a single group then one-way ANOVA was used to test overall differences between group means. If an overall test was significant, pairwise differences were tested using the Tukey HSD test. Group means were presented as mean ± standard error. Experiments were performed using HBECs from 3 or more donors on 3 or more separate occasions unless otherwise noted. P-values ≤ 0.05 were considered significant. Analyses were performed in SAS 9.3 (SAS Institute).

## Additional Information

**How to cite this article:** Ghosh, A. *et al*. Little cigars are more toxic than cigarettes and uniquely change the airway Gene and Protein Expression. *Sci. Rep.* **7**, 46239; doi: 10.1038/srep46239 (2017).

**Publisher's note:** Springer Nature remains neutral with regard to jurisdictional claims in published maps and institutional affiliations.

## Supplementary Material

Supplementary Material
