# Supplementary material for: Little Cigars are More Toxic than Cigarettes and Uniquely Change the Airway Gene and Protein Expression
Source: Sci Rep. 2017 Apr 27;7:46239. doi: 10.1038/srep46239 (PMC5406835; doi:10.1038/srep46239)
Supplement: Supplementary Material [file srep46239-s1.docx]

Supplementary figures for

**Little Cigars are More Toxic than Cigarettes and Uniquely Change the Airway Gene and Protein Expression**

## Arunava Ghosh, Sabri H. Abdelwahab, Steven L. Reeber, Boris Reidel, Abigail J. Marklew, Andrew J. Garrison, Shernita Lee, Hong Dang, Amy H. Herring, Gary L. Glish, Mehmet Kesimer & Robert Tarran

Corresponding author: [robert_tarran@med.unc.edu](mailto:robert_tarran@med.unc.edu)

## Supplementary figure 1

**
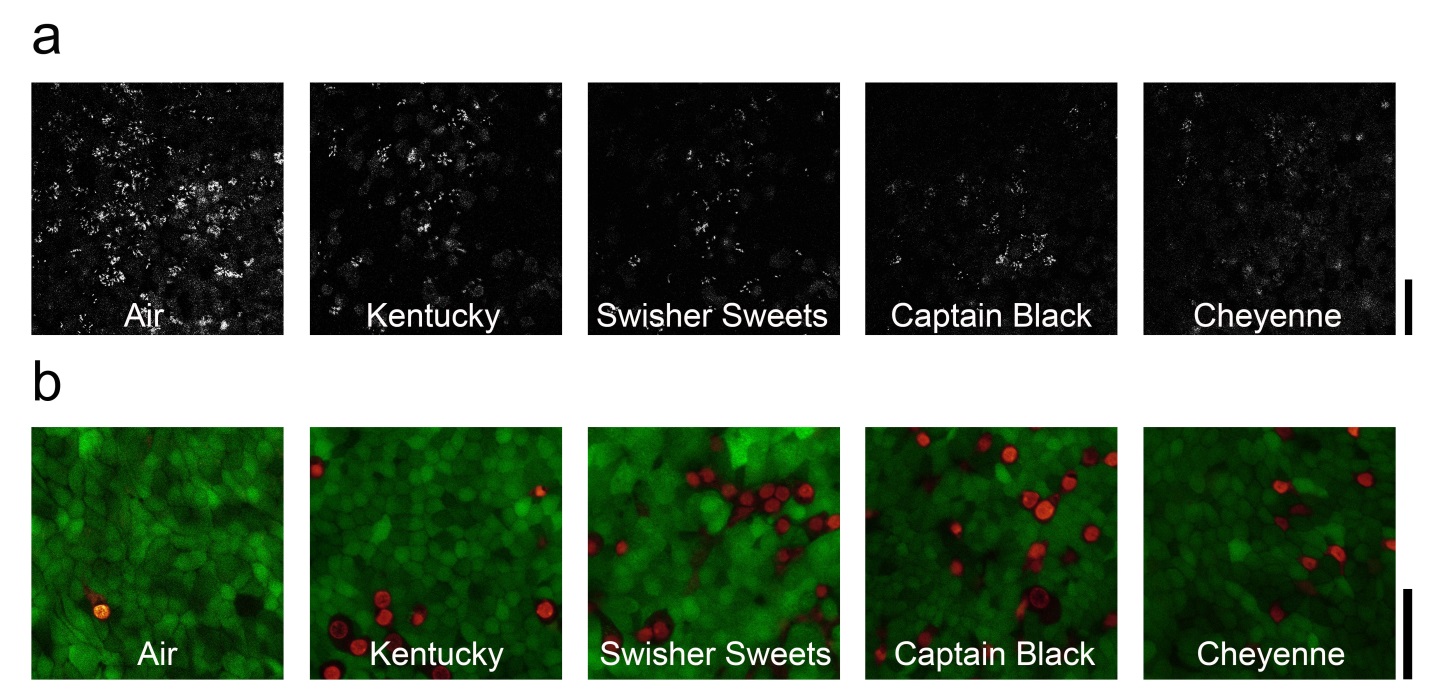
**

**Supplementary figure 1. Chronic little cigar exposure caused decreased apical ciliary abundance and increased cell death. (a)** Representative images of apical cilia distribution in chronic smoke and air exposed HBEC cultures. **(b)** Representative images of propidium iodide (red) uptake by chronic smoke exposed HBECs with calcein-AM staining (green) for live cells after chronic smoke exposure. Each field had on average 220-230 cells. Scale Bar is 50 µm.

**Supplementary figure 2**


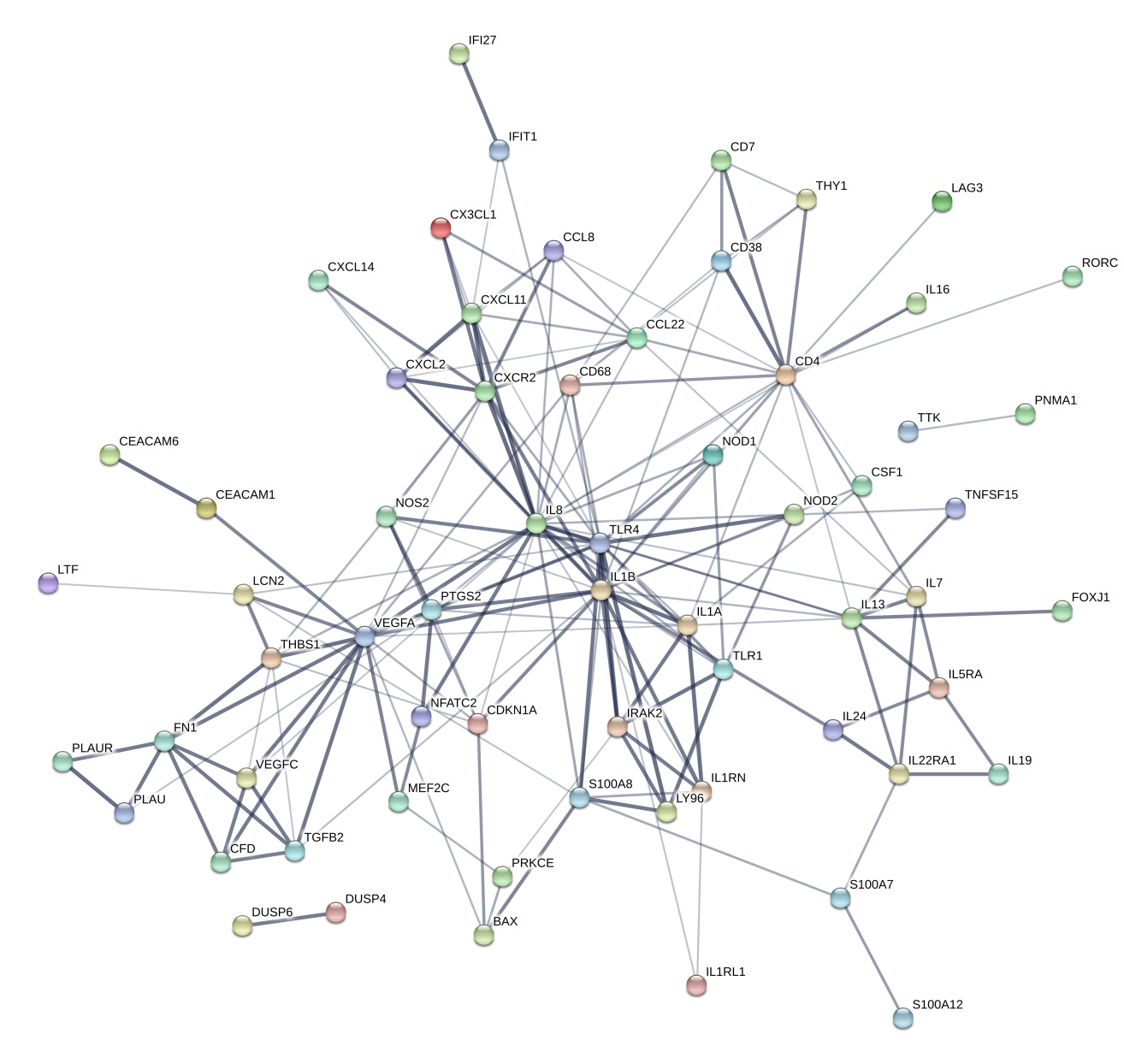


**Supplementary figure 2.** **Gene network showing interactions among the genes altered in Kentucky exposure compared to air.** Polarized HBECs were exposed to the smoke from 1 Kentucky research cigarette every day for 5 days (See Methods) and then RNA was collected for Nanostring analysis using the Pan-Cancer-Immune gene array. Samples were obtained from 3 individual donors. Pathway analysis was performed using String.

**Supplementary figure 3**


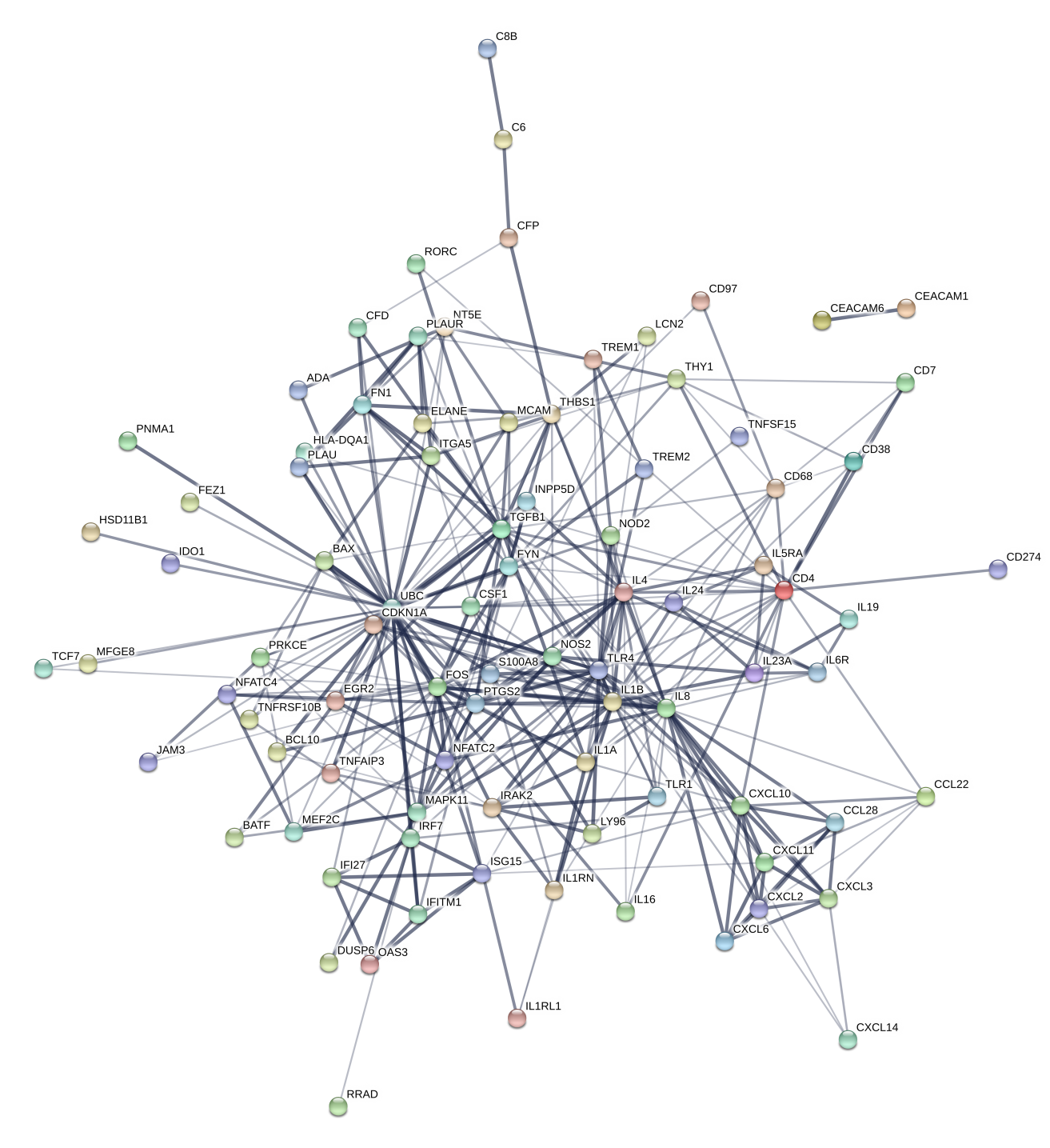


**Supplementary figure 3.** **Gene network showing interactions among the genes altered following Swisher Sweets little cigar exposure compared to air.** Polarized HBECs were exposed to the smoke from 1 Swisher Sweet little cigar every day for 5 days (See Methods) and then RNA was collected for Nanostring analysis using the Pan-Cancer-Immune gene array. Samples were obtained from 3 individual donors. Pathway analysis was performed using String.

**Supplementary figure 4**


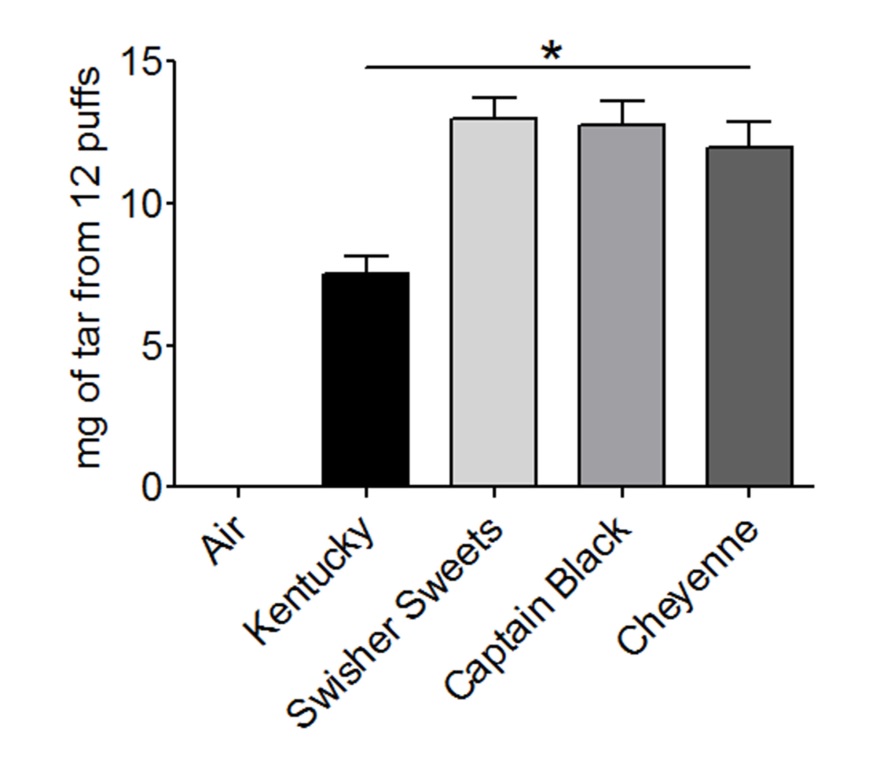


**Supplementary figure 4. Little Cigars produces more tar particles than Kentucky.** Cambridge filter pads were weighed using a Cahn microbalance and inserted in the line of a Borgwaldt smoke engine. 12 x 35 ml puffs were generated for the tobacco products listed and the filter pads were reweighed. The difference in weight was used as an indicator of the tar deposited from the smoke exposure. All the little cigars showed significantly increased (p<0.05) tar deposition compared to Kentucky research cigarettes. All data is n = 4.

**Supplemental Table 1.** **List of genes changed in HBECs after 5 days of exposure to Kentucky research cigarettes compared to air exposure.** Samples were probed with the NanoString PanCancer Immune panel of 770 genes. Criteria for significance were (i) fold change ≥ ± 2 and (ii) q-value ≤ 0.1. Data shown as mean gene expression. HBECs derived from 3 individual donors were used for the analysis.

| **Gene name** | **Air mean** | **Kentucky mean** | **Log_2_ fold change** | **p-value** | **q-value** |
| --- | --- | --- | --- | --- | --- |
| RORC | 379.843 | 79.520 | -2.256 | 0.000E+00 | 0.000E+00 |
| DMBT1 | 205.387 | 50.292 | -2.030 | 0.000E+00 | 0.000E+00 |
| TGFB2 | 180.352 | 61.704 | -1.547 | 0.000E+00 | 0.000E+00 |
| DUSP4 | 732.673 | 1596.370 | 1.124 | 0.000E+00 | 0.000E+00 |
| VEGFA | 525.050 | 1161.124 | 1.145 | 0.000E+00 | 0.000E+00 |
| THBD | 223.481 | 552.671 | 1.306 | 0.000E+00 | 0.000E+00 |
| TNFRSF12A | 183.550 | 517.765 | 1.496 | 0.000E+00 | 0.000E+00 |
| IL1RN | 387.544 | 1633.364 | 2.075 | 0.000E+00 | 0.000E+00 |
| THBS1 | 199.038 | 872.295 | 2.132 | 0.000E+00 | 0.000E+00 |
| CXCL14 | 201.045 | 1058.489 | 2.396 | 0.000E+00 | 0.000E+00 |
| IRAK2 | 68.866 | 365.126 | 2.407 | 0.000E+00 | 0.000E+00 |
| IL8 | 1202.718 | 13030.646 | 3.438 | 0.000E+00 | 0.000E+00 |
| LIF | 22.762 | 354.682 | 3.962 | 0.000E+00 | 0.000E+00 |
| IL1A | 13.307 | 231.159 | 4.119 | 0.000E+00 | 0.000E+00 |
| IL1B | 9.613 | 192.507 | 4.324 | 0.000E+00 | 0.000E+00 |
| TLR1 | 534.162 | 263.462 | -1.020 | 4.441E-16 | 2.026E-14 |
| CX3CL1 | 683.842 | 287.828 | -1.248 | 1.221E-15 | 5.244E-14 |
| PTGS2 | 11.766 | 259.732 | 4.464 | 2.331E-15 | 9.455E-14 |
| CDKN1A | 1832.647 | 6153.126 | 1.747 | 3.553E-15 | 1.365E-13 |
| LY96 | 48.115 | 122.910 | 1.353 | 9.992E-15 | 3.647E-13 |
| DUSP6 | 148.483 | 775.841 | 2.385 | 1.095E-13 | 3.632E-12 |
| BATF | 37.561 | 100.654 | 1.422 | 2.301E-13 | 7.000E-12 |
| CD7 | 5.773 | 39.720 | 2.783 | 3.512E-13 | 1.025E-11 |
| CSF1 | 48.861 | 7.649 | -2.675 | 7.453E-13 | 2.015E-11 |
| PRKCE | 162.227 | 71.966 | -1.173 | 2.031E-12 | 5.294E-11 |
| CFD | 146.864 | 55.243 | -1.411 | 5.118E-12 | 1.288E-10 |
| TLR4 | 90.047 | 24.773 | -1.862 | 5.320E-12 | 1.294E-10 |
| FOXJ1 | 11206.345 | 2328.330 | -2.267 | 2.120E-11 | 4.553E-10 |
| PLAU | 601.544 | 2049.337 | 1.768 | 5.445E-11 | 1.136E-09 |
| FN1 | 26.682 | 0.436 | -5.934 | 6.800E-11 | 1.379E-09 |
| LCN2 | 8430.471 | 18420.676 | 1.128 | 8.456E-11 | 1.668E-09 |
| NOD2 | 51.728 | 10.173 | -2.346 | 8.809E-11 | 1.692E-09 |
| VEGFC | 74.551 | 27.345 | -1.447 | 4.277E-10 | 7.806E-09 |
| S100A7 | 180.618 | 393.094 | 1.122 | 7.600E-10 | 1.353E-08 |
| TNFSF15 | 32.935 | 3.278 | -3.329 | 1.068E-09 | 1.779E-08 |
| RRAD | 262.670 | 28.614 | -3.198 | 1.072E-09 | 1.779E-08 |
| PLAUR | 165.156 | 341.760 | 1.049 | 1.686E-09 | 2.618E-08 |
| NFATC2 | 162.120 | 69.879 | -1.214 | 2.011E-09 | 3.058E-08 |
| IFIT1 | 183.966 | 90.144 | -1.029 | 8.475E-09 | 1.167E-07 |
| TNFRSF10C | 55.698 | 307.681 | 2.466 | 1.576E-08 | 2.092E-07 |
| S100A8 | 2831.992 | 13730.085 | 2.277 | 1.780E-08 | 2.321E-07 |
| IL19 | 114.218 | 876.714 | 2.940 | 2.695E-08 | 3.393E-07 |
| PNMA1 | 466.310 | 195.305 | -1.256 | 2.903E-08 | 3.592E-07 |
| MEF2C | 38.555 | 8.672 | -2.153 | 3.336E-08 | 4.059E-07 |
| CEACAM6 | 4267.765 | 12824.773 | 1.587 | 1.043E-07 | 1.171E-06 |
| SPA17 | 7022.224 | 2372.900 | -1.565 | 1.175E-07 | 1.299E-06 |
| BAX | 854.945 | 1834.849 | 1.102 | 1.762E-07 | 1.920E-06 |
| IL24 | 0.000 | 14.139 | 22.650 | 3.335E-07 | 3.528E-06 |
| INPP5D | 76.915 | 259.157 | 1.752 | 5.065E-07 | 5.208E-06 |
| CEACAM1 | 94.397 | 257.277 | 1.447 | 6.357E-07 | 6.445E-06 |
| IL5RA | 387.895 | 70.889 | -2.452 | 8.542E-07 | 8.426E-06 |
| S100A12 | 46.649 | 237.659 | 2.349 | 1.016E-06 | 9.763E-06 |
| CCL8 | 0.000 | 11.748 | 26.093 | 1.189E-06 | 1.112E-05 |
| NOS2A | 16.225 | 0.000 | -84.048 | 1.684E-06 | 1.537E-05 |
| CXCL2 | 320.133 | 647.479 | 1.016 | 5.987E-06 | 5.141E-05 |
| THY1 | 0.000 | 15.086 | 17.348 | 6.734E-06 | 5.650E-05 |
| CD4 | 238.129 | 77.518 | -1.619 | 7.398E-06 | 6.068E-05 |
| CD68 | 111.838 | 260.496 | 1.220 | 7.684E-06 | 6.233E-05 |
| IFI27 | 890.075 | 1785.013 | 1.004 | 1.375E-05 | 1.027E-04 |
| LAG3 | 15.467 | 0.000 | -21.082 | 1.687E-05 | 1.244E-04 |
| CCL15 | 44.651 | 20.092 | -1.152 | 4.591E-05 | 3.192E-04 |
| CCL22 | 11.111 | 30.743 | 1.468 | 6.584E-05 | 4.450E-04 |
| NOD1 | 42.789 | 20.069 | -1.092 | 1.346E-04 | 8.508E-04 |
| CD38 | 224.706 | 50.480 | -2.154 | 1.513E-04 | 9.242E-04 |
| C6 | 52.654 | 19.895 | -1.404 | 1.919E-04 | 1.130E-03 |
| SYCP1 | 0.000 | 8.979 | 23.126 | 2.723E-04 | 1.553E-03 |
| IL7 | 21.537 | 5.503 | -1.969 | 2.886E-04 | 1.633E-03 |
| CCRL2 | 3.723 | 17.740 | 2.252 | 3.034E-04 | 1.704E-03 |
| IL16 | 21.730 | 6.185 | -1.813 | 3.863E-04 | 2.136E-03 |
| IL1RL1 | 23.637 | 86.989 | 1.880 | 3.861E-04 | 2.136E-03 |
| CXCR2 | 26.465 | 10.215 | -1.373 | 4.534E-04 | 2.488E-03 |
| TTK | 75.901 | 37.695 | -1.010 | 4.935E-04 | 2.669E-03 |
| CXCL11 | 1.415 | 12.846 | 3.182 | 6.127E-04 | 3.218E-03 |
| IL13 | 1.857 | 13.760 | 2.890 | 6.667E-04 | 3.477E-03 |
| HSD11B1 | 3.125 | 16.370 | 2.389 | 8.201E-04 | 4.216E-03 |
| TARP | 0.000 | 9.731 | 15.934 | 1.053E-03 | 5.230E-03 |
| IL22RA1 | 18.414 | 5.526 | -1.737 | 1.453E-03 | 7.026E-03 |
| LTF | 7.760 | 0.000 | -36.354 | 1.687E-03 | 8.100E-03 |
| TNFRSF13C | 6.001 | 0.000 | -26.185 | 2.775E-03 | 1.264E-02 |
| IL2RB | 9.086 | 0.000 | -23.226 | 3.016E-03 | 1.359E-02 |
| TNFSF11 | 0.381 | 8.066 | 4.403 | 3.042E-03 | 1.362E-02 |
| SELPLG | 6.679 | 0.000 | -20.974 | 5.076E-03 | 2.167E-02 |
| IL34 | 9.495 | 0.660 | -3.847 | 5.065E-03 | 2.167E-02 |
| BAGE | 0.162 | 6.301 | 5.285 | 5.330E-03 | 2.262E-02 |
| IL26 | 4.876 | 15.322 | 1.652 | 5.628E-03 | 2.348E-02 |
| OSM | 0.000 | 6.894 | 19.488 | 5.617E-03 | 2.348E-02 |
| IDO1 | 71.491 | 190.425 | 1.413 | 5.788E-03 | 2.374E-02 |
| HLA-DRB4 | 8.014 | 18.808 | 1.231 | 6.456E-03 | 2.618E-02 |
| IFNG | 0.000 | 5.695 | 25.132 | 6.892E-03 | 2.780E-02 |
| JAM3 | 14.897 | 5.125 | -1.540 | 9.948E-03 | 3.802E-02 |
| C4B | 22.968 | 9.448 | -1.282 | 1.105E-02 | 4.156E-02 |
| IL12RB1 | 0.000 | 6.230 | 24.376 | 1.218E-02 | 4.561E-02 |
| CXCR4 | 4.580 | 0.000 | -22.010 | 1.375E-02 | 5.096E-02 |
| CD6 | 3.799 | 0.000 | -59.520 | 1.487E-02 | 5.411E-02 |
| HCK | 3.448 | 0.000 | -24.861 | 1.590E-02 | 5.747E-02 |
| FPR2 | 3.901 | 0.000 | -24.459 | 1.620E-02 | 5.826E-02 |
| LTK | 5.101 | 0.000 | -21.896 | 1.650E-02 | 5.903E-02 |
| APOE | 3.718 | 0.000 | -357.498 | 1.697E-02 | 5.961E-02 |
| TNFRSF17 | 0.000 | 5.271 | 19.537 | 2.378E-02 | 8.038E-02 |
| FAS | 12.194 | 3.809 | -1.679 | 2.530E-02 | 8.434E-02 |
| CFP | 0.000 | 6.148 | 44.606 | 2.577E-02 | 8.512E-02 |
| CCL26 | 4.909 | 0.000 | -68.859 | 2.732E-02 | 8.888E-02 |
| CD40LG | 0.000 | 6.295 | 21.254 | 2.739E-02 | 8.888E-02 |
| ELANE | 0.000 | 4.403 | 19.200 | 2.980E-02 | 9.574E-02 |

**Supplemental Table 2.** List of genes changed in HBECs after 5 days of exposure to Swisher Sweets little cigars compared to air (control). Samples were probed with the NanoString PanCancer Immune panel of 770 genes. Criteria for significance were (i) fold change ≥ ± 2 and (ii) q-value ≤ 0.1. Data shown as mean gene expression and HBECs from 3 individual donors were used for the analysis.

| **Gene name** | **Air mean** | **Little cigar mean** | **Log_2_ fold change** | **p-value** | **q-value** |
| --- | --- | --- | --- | --- | --- |
| CXCR4 | 4.580 | 0.000 | -55.682 | 2.804E-02 | 7.843E-02 |
| CD6 | 3.798 | 0.000 | -52.781 | 1.053E-02 | 3.400E-02 |
| NOS2A | 16.226 | 0.000 | -27.717 | 5.859E-08 | 4.152E-07 |
| TNFRSF13C | 6.001 | 0.000 | -26.870 | 2.627E-02 | 7.434E-02 |
| CD53 | 2.326 | 0.000 | -26.202 | 1.344E-02 | 4.211E-02 |
| HCK | 3.448 | 0.000 | -25.934 | 3.209E-02 | 8.807E-02 |
| IL16 | 21.729 | 0.796 | -4.771 | 9.116E-08 | 6.399E-07 |
| FN1 | 26.682 | 2.025 | -3.720 | 2.714E-09 | 2.573E-08 |
| IL5RA | 387.897 | 44.169 | -3.135 | 4.440E-10 | 4.767E-09 |
| RRAD | 262.673 | 30.749 | -3.095 | 1.345E-09 | 1.327E-08 |
| FOXJ1 | 11208.958 | 1476.039 | -2.925 | 0.000E+00 | 0.000E+00 |
| CD38 | 224.660 | 31.552 | -2.832 | 9.167E-07 | 5.624E-06 |
| DMBT1 | 205.373 | 32.779 | -2.647 | 0.000E+00 | 0.000E+00 |
| JAM3 | 14.897 | 2.406 | -2.631 | 5.108E-04 | 2.143E-03 |
| RORC | 379.852 | 67.746 | -2.487 | 0.000E+00 | 0.000E+00 |
| MEF2C | 38.555 | 6.894 | -2.484 | 5.601E-10 | 5.758E-09 |
| CD4 | 238.164 | 44.157 | -2.431 | 7.673E-11 | 9.493E-10 |
| C6 | 52.658 | 10.035 | -2.392 | 1.049E-08 | 8.799E-08 |
| SPA17 | 7022.700 | 1531.120 | -2.197 | 1.020E-13 | 1.817E-12 |
| CSF1 | 48.860 | 11.801 | -2.050 | 1.336E-10 | 1.573E-09 |
| CCL15 | 44.652 | 11.886 | -1.909 | 4.193E-09 | 3.733E-08 |
| LAG3 | 15.469 | 4.707 | -1.717 | 5.796E-03 | 1.977E-02 |
| CCL14 | 56.854 | 18.107 | -1.651 | 1.731E-08 | 1.404E-07 |
| PNMA1 | 466.249 | 151.052 | -1.626 | 1.128E-12 | 1.790E-11 |
| NOD2 | 51.728 | 17.374 | -1.574 | 5.149E-07 | 3.240E-06 |
| TLR4 | 90.048 | 31.602 | -1.511 | 3.863E-09 | 3.481E-08 |
| TCF7 | 81.703 | 29.456 | -1.472 | 4.698E-10 | 4.899E-09 |
| CFD | 146.863 | 53.618 | -1.454 | 5.230E-13 | 8.677E-12 |
| CCL28 | 105.159 | 38.594 | -1.446 | 2.033E-12 | 3.028E-11 |
| TNFSF15 | 32.937 | 12.100 | -1.445 | 8.220E-05 | 4.027E-04 |
| SMPD3 | 95.659 | 37.221 | -1.362 | 4.745E-11 | 6.185E-10 |
| FOS | 4930.835 | 1939.496 | -1.346 | 6.917E-11 | 8.706E-10 |
| HLA-DQA1 | 86.326 | 33.980 | -1.345 | 1.407E-04 | 6.626E-04 |
| NFATC2 | 162.121 | 63.963 | -1.342 | 5.234E-11 | 6.703E-10 |
| FYN | 39.093 | 15.549 | -1.330 | 1.080E-04 | 5.257E-04 |
| TNFAIP3 | 342.467 | 137.206 | -1.320 | 0.000E+00 | 0.000E+00 |
| IL3RA | 10.803 | 4.471 | -1.273 | 3.551E-02 | 9.623E-02 |
| TLR1 | 534.194 | 227.664 | -1.230 | 0.000E+00 | 0.000E+00 |
| CD97 | 1034.014 | 450.798 | -1.198 | 1.110E-16 | 2.614E-15 |
| KIT | 15.039 | 6.585 | -1.191 | 2.603E-02 | 7.423E-02 |
| IL7 | 21.532 | 9.860 | -1.127 | 8.375E-03 | 2.779E-02 |
| PRKCE | 162.225 | 78.622 | -1.045 | 1.734E-10 | 1.977E-09 |
| BCL10 | 1286.480 | 2591.232 | 1.010 | 1.110E-16 | 2.614E-15 |
| IRF7 | 59.272 | 120.508 | 1.024 | 8.143E-09 | 6.912E-08 |
| CDH5 | 25.513 | 53.411 | 1.066 | 5.263E-03 | 1.821E-02 |
| TNFRSF10B | 230.662 | 490.141 | 1.087 | 1.665E-15 | 3.684E-14 |
| DEFB1 | 7.120 | 15.346 | 1.108 | 2.689E-02 | 7.550E-02 |
| ICAM4 | 25.343 | 57.033 | 1.170 | 2.924E-04 | 1.293E-03 |
| CXCL3 | 145.567 | 334.718 | 1.201 | 1.101E-06 | 6.641E-06 |
| CXCL2 | 320.137 | 758.441 | 1.244 | 3.476E-08 | 2.587E-07 |
| BAX | 854.953 | 2027.624 | 1.246 | 2.762E-09 | 2.585E-08 |
| IL6R | 218.922 | 527.167 | 1.268 | 0.000E+00 | 0.000E+00 |
| UBC | 45271.056 | 111529.870 | 1.301 | 0.000E+00 | 0.000E+00 |
| BATF | 37.561 | 94.408 | 1.330 | 1.114E-11 | 1.505E-10 |
| TAP1 | 93.195 | 234.402 | 1.331 | 2.220E-16 | 5.065E-15 |
| CD79A | 4.871 | 12.415 | 1.350 | 3.643E-02 | 9.813E-02 |
| CXCL6 | 106.453 | 274.438 | 1.366 | 9.568E-07 | 5.821E-06 |
| MFGE8 | 265.920 | 687.943 | 1.371 | 1.813E-08 | 1.455E-07 |
| CXCL14 | 201.075 | 521.835 | 1.376 | 5.782E-12 | 8.276E-11 |
| TNFSF13B | 4.631 | 12.290 | 1.408 | 2.238E-02 | 6.509E-02 |
| NLRC5 | 9.593 | 25.486 | 1.410 | 5.655E-04 | 2.345E-03 |
| LCN2 | 8430.011 | 23143.595 | 1.457 | 1.110E-16 | 2.614E-15 |
| PLAUR | 165.158 | 458.817 | 1.474 | 0.000E+00 | 0.000E+00 |
| TGFB1 | 215.475 | 599.372 | 1.476 | 3.703E-13 | 6.286E-12 |
| IL23A | 11.603 | 32.319 | 1.478 | 2.590E-04 | 1.160E-03 |
| ADA | 43.267 | 120.701 | 1.480 | 2.442E-15 | 5.094E-14 |
| CD274 | 14.639 | 42.041 | 1.522 | 2.539E-07 | 1.700E-06 |
| ITGA5 | 99.788 | 289.443 | 1.536 | 0.000E+00 | 0.000E+00 |
| TNFRSF12A | 183.556 | 533.580 | 1.539 | 0.000E+00 | 0.000E+00 |
| NFATC4 | 30.091 | 88.305 | 1.553 | 2.243E-08 | 1.780E-07 |
| THBD | 223.483 | 669.703 | 1.583 | 0.000E+00 | 0.000E+00 |
| NT5E | 25.703 | 77.959 | 1.601 | 4.628E-05 | 2.362E-04 |
| ULBP2 | 32.287 | 98.164 | 1.604 | 1.110E-16 | 2.614E-15 |
| FEZ1 | 54.540 | 168.684 | 1.629 | 8.010E-13 | 1.299E-11 |
| LY96 | 48.112 | 155.088 | 1.689 | 0.000E+00 | 0.000E+00 |
| IFITM1 | 2102.588 | 7165.464 | 1.769 | 0.000E+00 | 0.000E+00 |
| CEACAM6 | 4267.996 | 15616.490 | 1.871 | 2.905E-10 | 3.213E-09 |
| OAS3 | 104.383 | 399.696 | 1.937 | 0.000E+00 | 0.000E+00 |
| THBS1 | 199.034 | 784.360 | 1.978 | 0.000E+00 | 0.000E+00 |
| CEACAM1 | 94.403 | 378.468 | 2.003 | 7.608E-12 | 1.048E-10 |
| EGR2 | 10.508 | 42.212 | 2.006 | 2.300E-08 | 1.806E-07 |
| TNFRSF11B | 2.527 | 10.337 | 2.032 | 1.111E-02 | 3.556E-02 |
| TREM2 | 5.869 | 24.347 | 2.052 | 1.712E-05 | 9.255E-05 |
| IRAK2 | 68.865 | 288.147 | 2.065 | 0.000E+00 | 0.000E+00 |
| IDO1 | 71.489 | 307.180 | 2.103 | 4.396E-05 | 2.260E-04 |
| CD68 | 111.840 | 496.785 | 2.151 | 2.331E-15 | 5.006E-14 |
| PLAU | 601.595 | 2881.775 | 2.260 | 1.110E-16 | 2.614E-15 |
| HSD11B1 | 3.124 | 15.028 | 2.266 | 6.610E-04 | 2.711E-03 |
| IL1RN | 387.550 | 1924.056 | 2.312 | 0.000E+00 | 0.000E+00 |
| CCRL2 | 3.723 | 19.062 | 2.356 | 6.691E-05 | 3.323E-04 |
| IL13 | 1.857 | 9.585 | 2.368 | 1.480E-02 | 4.560E-02 |
| CDKN1A | 1832.619 | 9485.547 | 2.372 | 0.000E+00 | 0.000E+00 |
| CCL22 | 11.112 | 59.295 | 2.416 | 2.665E-15 | 5.403E-14 |
| S100A8 | 2831.752 | 16202.746 | 2.516 | 4.245E-10 | 4.625E-09 |
| ISG15 | 220.517 | 1276.340 | 2.533 | 0.000E+00 | 0.000E+00 |
| INPP5D | 76.908 | 459.719 | 2.580 | 1.105E-13 | 1.920E-12 |
| CXCL10 | 5.740 | 35.063 | 2.611 | 3.862E-05 | 2.014E-04 |
| IFI27 | 890.118 | 5511.241 | 2.630 | 0.000E+00 | 0.000E+00 |
| IL19 | 114.219 | 726.936 | 2.670 | 1.213E-07 | 8.353E-07 |
| CD7 | 5.773 | 37.752 | 2.709 | 1.188E-12 | 1.846E-11 |
| S100A12 | 46.662 | 314.505 | 2.753 | 7.536E-09 | 6.472E-08 |
| TREM1 | 1.402 | 10.507 | 2.905 | 1.622E-03 | 6.265E-03 |
| DUSP6 | 148.480 | 1113.527 | 2.907 | 0.000E+00 | 0.000E+00 |
| TNFRSF10C | 55.702 | 433.536 | 2.960 | 5.213E-12 | 7.611E-11 |
| IL8 | 1202.853 | 12782.128 | 3.410 | 0.000E+00 | 0.000E+00 |
| MCAM | 1.670 | 18.975 | 3.506 | 1.912E-06 | 1.117E-05 |
| IL1RL1 | 23.628 | 274.347 | 3.537 | 4.672E-10 | 4.899E-09 |
| SELE | 0.292 | 5.222 | 4.161 | 1.783E-02 | 5.356E-02 |
| CXCL11 | 1.415 | 25.491 | 4.171 | 1.294E-09 | 1.294E-08 |
| LIF | 22.762 | 620.047 | 4.768 | 0.000E+00 | 0.000E+00 |
| PTGS2 | 11.766 | 356.219 | 4.920 | 0.000E+00 | 0.000E+00 |
| IL1A | 13.307 | 552.299 | 5.375 | 0.000E+00 | 0.000E+00 |
| IL1B | 9.616 | 514.081 | 5.740 | 0.000E+00 | 0.000E+00 |
| IL4 | 0.002 | 9.656 | 12.138 | 1.435E-03 | 5.601E-03 |
| KLRD1 | 0.000 | 5.105 | 16.236 | 1.496E-02 | 4.588E-02 |
| C1QB | 0.000 | 6.128 | 17.815 | 6.605E-03 | 2.212E-02 |
| CFP | 0.000 | 8.449 | 18.434 | 2.417E-03 | 8.956E-03 |
| MAGEC2 | 0.000 | 6.453 | 18.537 | 6.533E-03 | 2.208E-02 |
| AMICA1 | 0.000 | 10.497 | 19.683 | 1.369E-04 | 6.531E-04 |
| CD40LG | 0.000 | 5.006 | 20.429 | 2.128E-02 | 6.251E-02 |
| OSM | 0.000 | 4.357 | 20.714 | 3.658E-02 | 9.818E-02 |
| CHIT1 | 0.000 | 7.936 | 21.551 | 4.116E-03 | 1.459E-02 |
| ELANE | 0.000 | 7.918 | 21.637 | 6.897E-04 | 2.813E-03 |
| THY1 | 0.000 | 9.183 | 22.417 | 1.154E-03 | 4.555E-03 |
| CREB5 | 0.000 | 7.519 | 22.919 | 1.228E-02 | 3.880E-02 |
| TNFRSF17 | 0.000 | 4.477 | 23.827 | 2.316E-02 | 6.665E-02 |
| CD5 | 0.000 | 5.484 | 25.554 | 2.274E-02 | 6.588E-02 |
| LBP | 0.000 | 3.307 | 25.789 | 1.911E-02 | 5.693E-02 |
| TNFSF18 | 0.000 | 4.513 | 25.822 | 2.627E-02 | 7.434E-02 |
| CCL25 | 0.000 | 3.308 | 26.502 | 1.909E-02 | 5.693E-02 |
| C8B | 0.000 | 9.370 | 28.478 | 2.516E-04 | 1.141E-03 |
| IL24 | 0.000 | 19.185 | 31.626 | 2.176E-11 | 2.888E-10 |
| PASD1 | 0.000 | 3.245 | 41.689 | 3.081E-02 | 8.487E-02 |
| FCER2 | 0.000 | 4.084 | 74.747 | 2.319E-02 | 6.665E-02 |
| BTK | 0.000 | 4.068 | 133.779 | 1.198E-02 | 3.818E-02 |
| MAPK11 | 0.000 | 6.661 | 154.915 | 2.574E-04 | 1.160E-03 |

**Supplemental Table 3.** List of genes that were uniquely altered in HBECs after 5 days of exposure to Swisher Sweets little cigars relative to Kentucky research cigarettes. Samples were probed with the NanoString PanCancer Immune panel of 770 genes. Criteria for significance were (i) fold change ≥ ± 2 and (ii) q-value ≤0.1. Data shown as mean gene expression ± SEM. HBECs from 3 individual donors were used for the analysis.

| **Gene name** | **Kentucky mean** | **Little cigar mean** | **Log_2_ fold change** | **p-value** | **q-value** |
| --- | --- | --- | --- | --- | --- |
| IL12RB1 | 6.231 | 0.000 | -72.061 | 5.803E-03 | 5.724E-02 |
| CCL8 | 11.782 | 0.379 | -4.960 | 4.791E-04 | 8.531E-03 |
| CXCL14 | 1058.416 | 521.774 | -1.020 | 4.242E-08 | 2.581E-06 |
| IRF7 | 59.642 | 120.502 | 1.015 | 8.434E-08 | 4.398E-06 |
| TICAM2 | 27.195 | 57.029 | 1.068 | 2.189E-04 | 4.700E-03 |
| DDX58 | 106.087 | 231.725 | 1.127 | 3.673E-07 | 1.577E-05 |
| IFITM1 | 3129.769 | 7165.198 | 1.195 | 1.998E-15 | 2.918E-13 |
| CXCR2 | 10.215 | 24.169 | 1.242 | 3.851E-03 | 4.074E-02 |
| IL1A | 231.173 | 552.329 | 1.257 | 0.000E+00 | 0.000E+00 |
| TREM2 | 10.021 | 24.349 | 1.281 | 5.101E-03 | 5.101E-02 |
| IL1B | 192.506 | 514.049 | 1.417 | 2.969E-04 | 5.858E-03 |
| IFIT2 | 41.299 | 112.636 | 1.447 | 3.244E-13 | 3.383E-11 |
| OAS3 | 141.986 | 399.717 | 1.493 | 2.753E-13 | 3.350E-11 |
| IL23A | 10.974 | 32.320 | 1.558 | 6.197E-04 | 1.052E-02 |
| IFI27 | 1785.166 | 5511.016 | 1.626 | 3.197E-12 | 2.917E-10 |
| IL1RL1 | 86.973 | 274.332 | 1.657 | 2.028E-03 | 2.597E-02 |
| ISG15 | 400.652 | 1276.297 | 1.672 | 5.551E-16 | 1.351E-13 |
| NT5E | 21.341 | 77.957 | 1.869 | 8.468E-06 | 2.576E-04 |
| EGR2 | 11.096 | 42.211 | 1.928 | 5.238E-07 | 2.124E-05 |
| IFIT1 | 90.149 | 367.200 | 2.026 | 0.000E+00 | 0.000E+00 |
| FAS | 3.810 | 17.267 | 2.180 | 1.783E-03 | 2.456E-02 |
| DEFB1 | 2.513 | 15.347 | 2.610 | 5.438E-04 | 9.452E-03 |
| MCAM | 2.167 | 18.975 | 3.130 | 9.163E-05 | 2.306E-03 |
| CXCL10 | 0.000 | 35.059 | 18.950 | 3.555E-07 | 1.577E-05 |
| APOE | 0.000 | 4.932 | 21.035 | 1.115E-02 | 9.251E-02 |
| CCL26 | 0.000 | 8.007 | 21.378 | 2.657E-03 | 3.180E-02 |
| CREB5 | 0.000 | 7.519 | 21.431 | 6.226E-03 | 5.856E-02 |
| SELPLG | 0.000 | 12.040 | 26.846 | 2.568E-04 | 5.206E-03 |
| AIRE | 0.000 | 4.066 | 27.232 | 1.023E-02 | 8.680E-02 |
| JAK3 | 0.000 | 5.529 | 47.640 | 4.892E-03 | 4.960E-02 |
| TREM1 | 0.000 | 10.507 | 77.188 | 1.385E-04 | 3.160E-03 |
| MAPK11 | 0.000 | 6.662 | 94.122 | 3.009E-03 | 3.543E-02 |

**Supplemental Table 4.**  List of proteins significantly altered in HBEC ASL after chronic (5 day) exposure to air (control), Kentucky research cigarettes or little cigars. The mean of the total precursor ion intensity with p-value<0.005, as determined by ANOVA, is shown.

| Accession Number | p-Value | Air Mean | Kentucky Mean | Cheyenne Mean | Captain Black Mean | Swisher Sweets Mean |
| --- | --- | --- | --- | --- | --- | --- |
| B2R5T2_HUMAN | 1.00E-04 | 15942017 | 68366667 | 2.17E+08 | 2.31E+08 | 1.78E+08 |
| Q59EP1_HUMAN | 1.10E-04 | 1.8E+08 | 3.87E+08 | 4.75E+08 | 4.05E+08 | 4.48E+08 |
| A8K8D9_HUMAN | 1.10E-04 | 8108917 | 28750000 | 39983333 | 42083333 | 45228167 |
| AK1C1_HUMAN | 1.20E-04 | 1.46E+08 | 4.33E+08 | 6.91E+08 | 5.81E+08 | 6.86E+08 |
| ECM1_HUMAN | 1.20E-04 | 35800000 | 59633333 | 2.86E+08 | 2.64E+08 | 2.21E+08 |
| AT8B1_HUMAN | 1.20E-04 | 9738017 | 26966667 | 45600000 | 46233333 | 58133333 |
| V9HW42_HUMAN | 1.30E-04 | 9.73E+09 | 1.16E+10 | 1.51E+10 | 1.44E+10 | 1.61E+10 |
| ES8L2_HUMAN | 1.30E-04 | 5.6E+08 | 9.21E+08 | 1.36E+09 | 1.15E+09 | 1.43E+09 |
| GSHR_HUMAN | 1.50E-04 | 84250000 | 1.44E+08 | 2.6E+08 | 2.14E+08 | 1.69E+08 |
| ES8L1_HUMAN | 1.60E-04 | 2.7E+08 | 3.76E+08 | 6.33E+08 | 5.09E+08 | 6.37E+08 |
| G3P_HUMAN | 1.70E-04 | 5.17E+08 | 6.09E+08 | 7.79E+08 | 7.16E+08 | 7.51E+08 |
| SG3A1_HUMAN | 1.80E-04 | 3.85E+09 | 9.48E+08 | 2.14E+08 | 2.9E+08 | 3.8E+08 |
| MMP7_HUMAN | 1.80E-04 | 22057600 | 0 | 0 | 0 | 0 |
| BAIP2_HUMAN | 2.10E-04 | 5.34E+08 | 7.18E+08 | 7.9E+08 | 8.23E+08 | 9.76E+08 |
| A0A0G2JPR0_HUMAN | 2.10E-04 | 46900117 | 13038233 | 3216667 | 5645817 | 1726600 |
| BLVRB_HUMAN | 2.10E-04 | 2819833 | 16662133 | 28473233 | 30100000 | 27233333 |
| EF1A1_HUMAN | 2.20E-04 | 3.18E+08 | 5.76E+08 | 6.52E+08 | 5.59E+08 | 6.11E+08 |
| LEG3_HUMAN | 2.60E-04 | 3.63E+08 | 7.33E+08 | 1.12E+09 | 9.92E+08 | 7.85E+08 |
| ARF1_HUMAN | 2.80E-04 | 1.33E+08 | 2.43E+08 | 3.28E+08 | 2.8E+08 | 2.97E+08 |
| A0A087WWM1_HUMAN | 3.10E-04 | 5.72E+09 | 8.72E+09 | 1.14E+10 | 1.16E+10 | 1.37E+10 |
| A0A0C4DGG1_HUMAN | 3.10E-04 | 4927485 | 20883333 | 31103517 | 28783333 | 31883333 |
| Q3KRG8_HUMAN | 3.20E-04 | 1.63E+08 | 3.68E+08 | 1.03E+09 | 6.76E+08 | 8.72E+08 |
| Q53HG7_HUMAN | 3.30E-04 | 30000000 | 43650000 | 56283333 | 52150000 | 59450000 |
| RAB5C_HUMAN | 3.40E-04 | 53366667 | 77066667 | 97866667 | 1.06E+08 | 1.09E+08 |
| I1SRC5_HUMAN | 3.60E-04 | 40283333 | 53016667 | 83016667 | 87866667 | 79316667 |
| MOES_HUMAN | 3.90E-04 | 4.05E+09 | 4.66E+09 | 6.44E+09 | 5.96E+09 | 6.87E+09 |
| ITLN1_HUMAN | 3.90E-04 | 8823900 | 409683.3 | 0 | 0 | 0 |
| RADI_HUMAN | 4.30E-04 | 4.83E+09 | 5.57E+09 | 7.56E+09 | 7.1E+09 | 7.94E+09 |
| PTPRS_HUMAN | 4.40E-04 | 5913517 | 1589017 | 211533.3 | 982583.3 | 91543.33 |
| NHRF1_HUMAN | 4.90E-04 | 1.68E+09 | 2.54E+09 | 2.84E+09 | 2.89E+09 | 3.14E+09 |
| MYOF_HUMAN | 4.90E-04 | 1.65E+08 | 3.44E+08 | 3.94E+08 | 3.75E+08 | 3.96E+08 |
| B4E0Y9_HUMAN | 5.00E-04 | 56033333 | 1.03E+08 | 1.38E+08 | 1.18E+08 | 1.44E+08 |
| RS27A_HUMAN | 5.10E-04 | 1.99E+08 | 2.87E+08 | 4.72E+08 | 4.86E+08 | 4.33E+08 |
| SPON2_HUMAN | 5.10E-04 | 1.34E+08 | 45100000 | 19184600 | 37576000 | 32583333 |
| B2RA03_HUMAN | 5.20E-04 | 1.2E+08 | 4.57E+08 | 6.67E+08 | 6.54E+08 | 3.8E+08 |
| SNP23_HUMAN | 5.20E-04 | 16816667 | 30400000 | 45283333 | 51550000 | 58266667 |
| B2R6S5_HUMAN | 6.30E-04 | 6024523 | 30853183 | 57966667 | 59836933 | 46583333 |
| A8K8Z4_HUMAN | 6.30E-04 | 5388283 | 2180850 | 724333.3 | 1828867 | 889266.7 |
| Q5JQ44_HUMAN | 6.60E-04 | 2150617 | 17788650 | 38684150 | 34718667 | 30066667 |
| CTL4_HUMAN | 7.70E-04 | 2.88E+08 | 3.56E+08 | 4.23E+08 | 4.26E+08 | 4.8E+08 |
| Q53FK3_HUMAN | 7.80E-04 | 36533333 | 73150000 | 2E+08 | 1.52E+08 | 1.99E+08 |
| CBR1_HUMAN | 9.00E-04 | 25950000 | 55850000 | 56650000 | 47866667 | 53433333 |
| F6KPG5_HUMAN | 9.50E-04 | 4.64E+09 | 5.84E+08 | 1.94E+09 | 1.29E+09 | 1.19E+09 |
| 1433Z_HUMAN | 1.00E-03 | 7.81E+08 | 9.95E+08 | 1.27E+09 | 1.26E+09 | 1.31E+09 |
| FUCO_HUMAN | 1.00E-03 | 1.06E+08 | 1.9E+08 | 3.32E+08 | 3.17E+08 | 3.24E+08 |
| GNA11_HUMAN | 1.10E-03 | 2.22E+08 | 3.43E+08 | 4.11E+08 | 4.25E+08 | 4.33E+08 |
| KLK10_HUMAN | 1.10E-03 | 5.87E+08 | 1.12E+09 | 6.41E+08 | 7.87E+08 | 8.19E+08 |
| GSLG1_HUMAN | 1.10E-03 | 17616667 | 22333333 | 50683333 | 43950000 | 37366667 |
| A0A024RC87_HUMAN | 1.10E-03 | 6853950 | 25283333 | 20466667 | 23166667 | 24150000 |
| SARG_HUMAN | 1.40E-03 | 20643200 | 63450000 | 1.63E+08 | 1.43E+08 | 1.43E+08 |
| AL3A1_HUMAN | 1.50E-03 | 1.17E+09 | 2.69E+09 | 4.33E+09 | 4.1E+09 | 3.21E+09 |
| Q68CK4_HUMAN | 1.80E-03 | 4.41E+08 | 7E+08 | 7.88E+08 | 9.55E+08 | 9.07E+08 |
| SODC_HUMAN | 1.80E-03 | 1.26E+08 | 2.46E+08 | 3.97E+08 | 4.42E+08 | 3.11E+08 |
| A0A087WT12_HUMAN | 1.90E-03 | 23800000 | 23633333 | 37816667 | 36800000 | 39633333 |
| A0A024R462_HUMAN | 2.30E-03 | 4184733 | 0 | 0 | 0 | 0 |
| A0A024R872_HUMAN | 2.40E-03 | 60183333 | 95466667 | 1.22E+08 | 1.18E+08 | 1.2E+08 |
| PRDX2_HUMAN | 2.60E-03 | 3.4E+08 | 5.35E+08 | 7.12E+08 | 6.74E+08 | 5.94E+08 |
| CAB39_HUMAN | 2.70E-03 | 1.34E+08 | 2.07E+08 | 2.25E+08 | 2.27E+08 | 2.42E+08 |
| MA1C1_HUMAN | 2.80E-03 | 13440367 | 3564350 | 0 | 2092300 | 3604983 |
| RAB5B_HUMAN | 2.80E-03 | 26400000 | 36316667 | 47266667 | 60650000 | 50150000 |
| CLUS_HUMAN | 2.90E-03 | 4.85E+09 | 4.13E+09 | 2.55E+09 | 2.97E+09 | 3.31E+09 |
| THIO_HUMAN | 2.90E-03 | 6.61E+08 | 8.84E+08 | 1.22E+09 | 1.28E+09 | 9.74E+08 |
| B4E1P0_HUMAN | 2.90E-03 | 6447733 | 21916667 | 37183333 | 37915517 | 27550000 |
| BSSP4_HUMAN | 3.00E-03 | 2.15E+08 | 2.08E+08 | 3.2E+08 | 3.58E+08 | 3.54E+08 |
| TRFE_HUMAN | 3.30E-03 | 1.63E+09 | 59500000 | 1.6E+08 | 1.63E+08 | 1E+08 |
| BGH3_HUMAN | 3.30E-03 | 25488050 | 3983400 | 9273667 | 13569783 | 9024017 |
| STK24_HUMAN | 3.40E-03 | 69333333 | 1.39E+08 | 1.66E+08 | 1.43E+08 | 1.86E+08 |
| G9FP35_HUMAN | 3.40E-03 | 1.46E+08 | 2.62E+08 | 3.4E+08 | 3.61E+08 | 3.56E+08 |
| KPYM_HUMAN | 3.50E-03 | 9.79E+08 | 1.1E+09 | 1.38E+09 | 1.26E+09 | 1.35E+09 |
| STXB2_HUMAN | 3.50E-03 | 1.62E+08 | 2.35E+08 | 3.17E+08 | 2.95E+08 | 3.52E+08 |
| A8K2I7_HUMAN | 3.50E-03 | 15082550 | 39550000 | 45766667 | 50866667 | 53583333 |
| 1433G_HUMAN | 3.70E-03 | 2.07E+08 | 2.91E+08 | 4.79E+08 | 3.86E+08 | 4.27E+08 |
| K22E_HUMAN | 3.90E-03 | 4.36E+08 | 6.29E+08 | 2.22E+09 | 1.69E+09 | 9.21E+08 |
| ARL3_HUMAN | 3.90E-03 | 6070583 | 22000000 | 27266667 | 24590633 | 26643600 |
| CN37_HUMAN | 4.00E-03 | 21095750 | 44483333 | 76816667 | 62850000 | 62233333 |
| B4E324_HUMAN | 4.00E-03 | 45966667 | 63283333 | 1.28E+08 | 1.01E+08 | 1.21E+08 |
| B2ZDQ1_HUMAN | 4.10E-03 | 3.91E+09 | 5.14E+09 | 1.14E+10 | 9.38E+09 | 9.67E+09 |
| K1C9_HUMAN | 4.30E-03 | 2.93E+08 | 6.71E+08 | 1.31E+09 | 1.44E+09 | 7.99E+08 |
| GALT5_HUMAN | 4.30E-03 | 2800383 | 10825267 | 85050000 | 52317800 | 64050000 |
| Q4W4Y1_HUMAN | 4.40E-03 | 5.56E+08 | 7.97E+08 | 8.08E+08 | 8.29E+08 | 9.13E+08 |
| CI009_HUMAN | 4.60E-03 | 5791067 | 4001767 | 0 | 1192883 | 929033.3 |
| STOM_HUMAN | 4.70E-03 | 6.89E+08 | 8.56E+08 | 1.78E+09 | 1.51E+09 | 1.43E+09 |
| A0A024RE18_HUMAN | 4.80E-03 | 43250000 | 71716667 | 79150000 | 1.02E+08 | 99666667 |
| HSP7C_HUMAN | 4.90E-03 | 8E+08 | 9.89E+08 | 1.22E+09 | 1.18E+09 | 1.16E+09 |
| A8KAH3_HUMAN | 4.90E-03 | 855566.7 | 7133333 | 15500000 | 14660233 | 17147733 |
| CLIC1_HUMAN | 5.00E-03 | 1.23E+09 | 1.67E+09 | 2.19E+09 | 1.91E+09 | 2.26E+09 |

**Supplemental Table 5.** List of chemicals present in tar particle extract of different little cigars and Kentucky research cigarettes as determined by Gas Chromatography-Mass Spectrometry. +/- denote presence/absence respectively.

| **Compound** | **Kentucky** | **Swisher Sweets** | **Captain Black** | **Cheyenne** |
| --- | --- | --- | --- | --- |
| 1,2,3-butanetriol | + | + | + | + |
| 1,2,3-propanetriol, monoacetate | + | - | - | - |
| 1,2-hexadecanediol | - | + | + | + |
| 1,5-pentanediol | - | + | + | + |
| 2,3'-dipyridyl | + | + | + | + |
| 2,4-dihydroxybutanoic acid | - | + | + | + |
| 2-deoxy-D-erythro-pentitol | - | + | + | + |
| 2-furoic acid | - | + | + | + |
| 2-methylglyceric acid | - | + | + | + |
| 2-methyl-lactic acid | + | + | + | + |
| 2-thiobarbituric acid | - | + | + | + |
| 3,4,5-trihydroxypentanoic acid | - | - | + | - |
| 3-deoxy-D-arabino-hexonic acid lactone | - | + | - | + |
| 3-deoxypentonic acid | - | + | + | + |
| 3-ethylphenol | - | + | + | + |
| 3-furoic acid | - | + | + | - |
| 3-hydroxybenzoic acid | - | + | + | + |
| 3-hydroxypropionic acid | - | + | + | + |
| 3-methyl indole | + | + | + | - |
| 3-pyridinol | + | + | + | + |
| 4-methylcatechol | - | + | + | + |
| 4-methylvaleric acid | + | + | + | + |
| 4-pyridinol | + | + | + | + |
| 5-allyl-1-methoxy-2,3-dihydroxybenzene | - | + | + | + |
| 5-ethyl isatin | - | + | + | + |
| 6-deoxy-D-glucitol | - | + | + | + |
| 6-methyl-3-pyridinol | - | + | + | + |
| acetic acid | + | + | - | - |
| acrylic acid | + | + | + | + |
| alpha-linolenic acid | - | + | + | + |
| ammonia | - | + | - | - |
| arabinofuranose (arabinose) | + | + | + | + |
| arabino-hexos-2-ulose | - | + | + | + |
| arabitol | - | + | + | + |
| benzoic acid | - | + | + | + |
| beta-D-galactofuranose | + | + | + | + |
| beta-hydroxybutyric acid | - | + | + | + |
| caproic acid | + | + | + | + |
| caprylic acid | - | + | + | + |
| catechol | - | + | + | + |
| cotinine | + | + | + | + |
| coumaran (2,3-dihydrobenzofuran) | + | - | - | - |
| crotonic acid | + | + | + | + |
| D-fructose | + | - | - | - |
| diethylene glycol | + | - | - | + |
| diethylene glycol dibenzoate | + | + | - | - |
| diethylene glycol dimethyl ether | + | + | + | + |
| dihydro-3,4-hydroxy-2(3H)-furanone | - | + | + | + |
| dihydroxyacetone | - | + | + | - |
| docosanoic acid (behenic acid) | - | + | + | + |
| eicosanoic acid (arachidic acid) | - | + | + | + |
| erythritol | - | + | + | + |
| ethanol | + | + | + | + |
| ethyl vanillin | - | - | + | - |
| ethylene glycol | - | + | + | + |
| floridoside (2-O-glycerol-.alpha.-D-galactopyranoside) | + | + | + | + |
| formic acid | + | + | + | + |
| gamma-hydroxyvaleric acid | - | - | + | + |
| glyceric acid | - | + | + | + |
| glycerin | + | - | - | - |
| glycerol | + | + | + | + |
| glycolic acid | + | + | + | + |
| heptacosane | + | - | + | - |
| heptadecanoic acid (margaric acid) | - | + | + | + |
| indole | + | + | + | - |
| inositol | + | + | + | + |
| isonicotinic acid | - | + | + | + |
| isovaleric acid | - | + | + | + |
| lactic acid | - | + | + | + |
| levoglucosan | + | + | + | + |
| levulinic acid | - | + | + | + |
| linoleic acid | - | + | + | + |
| malic acid | - | + | + | - |
| m-cresol | - | + | + | + |
| methylhydroquinone | - | + | + | + |
| myosmine | + | - | + | - |
| nicotine | + | + | + | + |
| nicotinic acid (niacin) | - | + | + | + |
| N-propylbenzamide | + | + | - | - |
| o-cresol | - | + | + | + |
| oleamide | + | + | + | + |
| o-toluic acid | - | + | + | + |
| palmitic acid | + | + | + | + |
| pentadecanoic acid | - | + | + | + |
| phenol | - | + | + | + |
| phenylacetic acid | - | + | + | + |
| phyt-2-ene (3,7,11,15-tetramethyl-2-hexadcene) | + | - | + | - |
| phytol | + | + | + | + |
| pinene | + | + | + | + |
| pipecolic acid | - | + | + | - |
| propanol | + | + | + | + |
| propionic acid | + | + | + | + |
| propylene glycol | + | + | + | + |
| pyrogallol | - | + | + | + |
| pyruvic acid | - | + | + | + |
| ribitol | - | + | + | + |
| sorbose | + | - | - | - |
| squalene | - | - | - | + |
| stearic acid | + | + | + | + |
| succinic acid | - | + | + | + |
| threonic acid | - | + | + | + |
| toluene | + | - | - | - |
| triacetin | + | + | + | + |
| Vanillyl glycol | - | - | - | + |
| xylitol | - | + | + | + |
| xylonic acid, delta lactone | - | - | + | - |
